# Explainable uncertainty quantifications for deep learning-based molecular property prediction

**DOI:** 10.1186/s13321-023-00682-3

**Published:** 2023-02-03

**Authors:** Chu-I Yang, Yi-Pei Li

**Affiliations:** 1 Department of Chemical Engineering, National Taiwan University, No. 1, Sec. 4, Roosevelt Road, Taipei, 10617 Taiwan; 2 Taiwan International Graduate Program (TIGP), Academia Sinica, No. 128, Sec. 2, Academia Road, Taipei, 11529 Taiwan

**Keywords:** Explainable AI, Uncertainty quantifications, Deep learning, Molecular property prediction

## Abstract

Quantifying uncertainty in machine learning is important in new research areas with scarce high-quality data. In this work, we develop an explainable uncertainty quantification method for deep learning-based molecular property prediction. This method can capture aleatoric and epistemic uncertainties separately and attribute the uncertainties to atoms present in the molecule. The atom-based uncertainty method provides an extra layer of chemical insight to the estimated uncertainties, i.e., one can analyze individual atomic uncertainty values to diagnose the chemical component that introduces uncertainty to the prediction. Our experiments suggest that atomic uncertainty can detect unseen chemical structures and identify chemical species whose data are potentially associated with significant noise. Furthermore, we propose a post-hoc calibration method to refine the uncertainty quantified by ensemble models for better confidence interval estimates. This work improves uncertainty calibration and provides a framework for assessing whether and why a prediction should be considered unreliable.

**Graphical Abstract:**

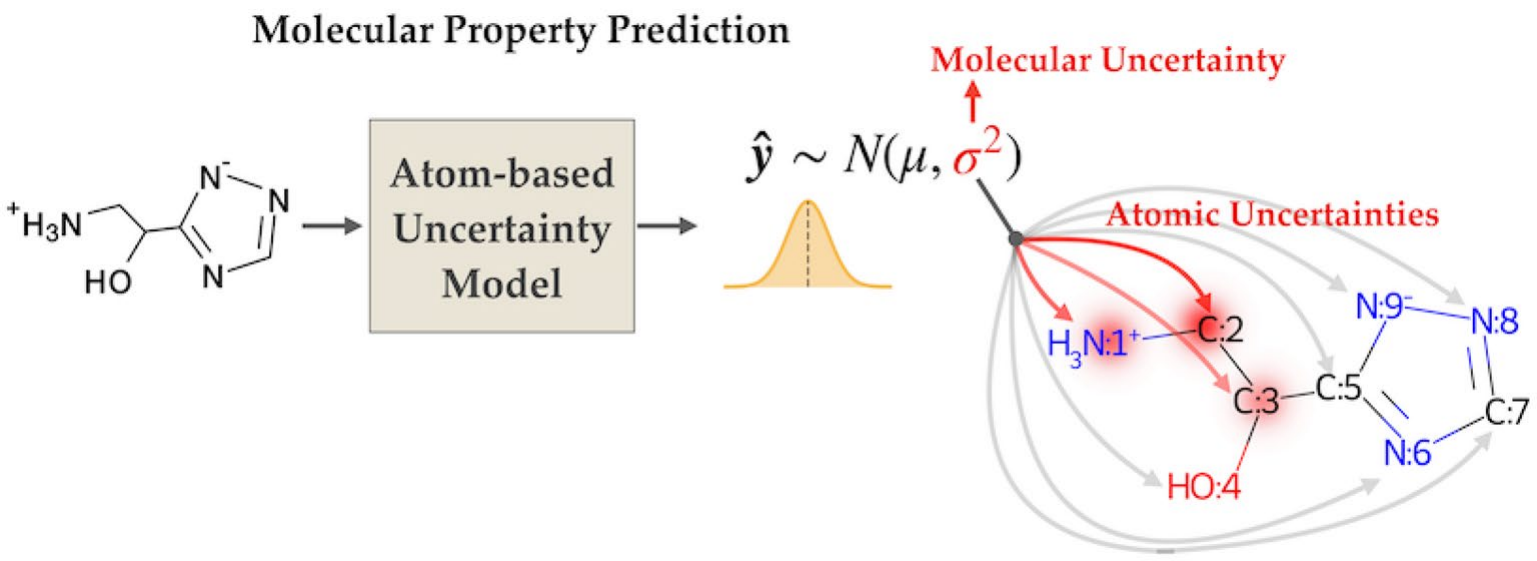

**Supplementary Information:**

The online version contains supplementary material available at 10.1186/s13321-023-00682-3.

## Introduction

With recent advances in deep neural networks (DNNs), machine learning has been widely applied in molecular property prediction and has successfully facilitated the development pipelines in many different applications [1, 2], including drug design [3], chemical biology [4], retrosynthesis [5, 6], and reaction engineering [7]. However, the key to the success of machine learning is comprehensive and high-quality datasets, which can be challenging to obtain in some areas of chemistry. Though large amounts of chemical data have been accumulated in literature over the years, the heterogeneous quality of data derived from different sources can significantly impact the harmonization of information and, hence, influence model performance [8]. Moreover, given that research is performed with a clearly defined goal and question in mind, the data distributions in the literature usually focus on certain regions of chemical spaces, so the accuracy of data-driven models is not always satisfactory in new research fields [9]. Therefore, assessing when and to what extent a prediction can be considered reliable is crucial for applying machine learning in molecular property prediction, especially when targeting new chemicals that have not been investigated before [10].

**Fig. 1:**
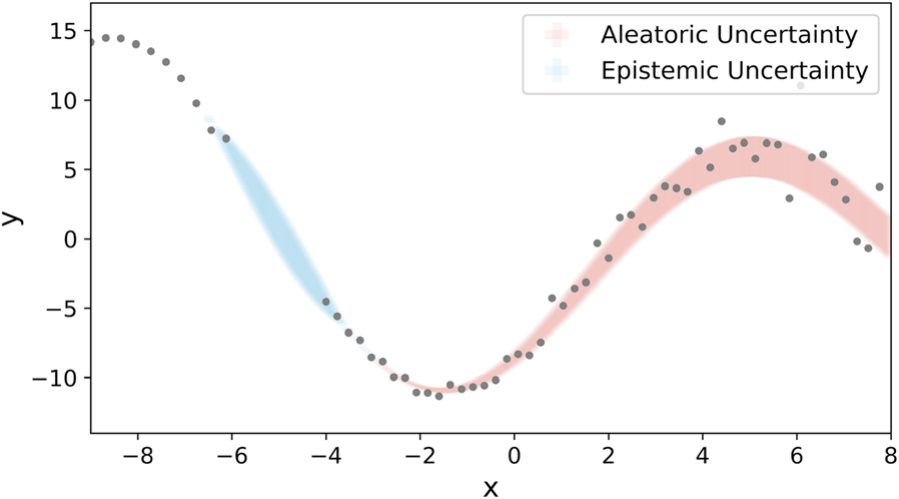
An illustration of the difference between aleatoric and epistemic uncertainties. The dots on the plot represent the available data points. Aleatoric uncertainty captures varying degrees of inherent noise in the data, while epistemic uncertainty reflects the ignorance gap due to a lack of data

Significant progress toward this end has been achieved by estimating the variance of predictions with uncertainty quantification methods [11–21]. In previous papers, Bayesian neural networks (BNN) have long been studied as an effective way to model uncertainty in a DNN prediction by treating weights and outputs as probability distributions [22, 23]. However, learning distributions over weights makes BNN more complicated to train and use than other neural networks. Therefore, Bayesian approximation methods such as Deep Ensembles [24], Monte Carlo dropout [25], Bayesian by Backprop [26], and Discriminative Jackknife [27] and conformal prediction methods such as Local Valid and Discriminative confidence intervals (LDV) [28] and Conformalized Quantile Regression (CQR) [29] have been proposed to quantify uncertainty in deep learning-based molecular property prediction [16, 28, 30]. These uncertainty quantification methods are designed to model either or both aleatoric and epistemic uncertainties [31–33], which refer to the irreducible and reducible parts of the uncertainty [32], respectively. In the context of molecular property prediction, aleatoric uncertainty usually refers to the output uncertainty induced by the inherent noise in the data caused by the limitation of the resolution of experimental techniques. When not explicitly modeled, aleatoric uncertainty is often assumed to be the same for all the samples (*homoscedastic* aleatoric uncertainty) [31, 34]. However, this assumption is not always true because, in chemistry applications, one often needs to collect data from multiple sources of different accuracy, which leads to a data-dependent aleatoric uncertainty and hence requires determining the degree of uncertainty in each datapoint (*heteroscedastic* aleatoric uncertainty) [31, 34]. On the other hand, epistemic uncertainty refers to the uncertainty arising from distributions over model parameters. In principle, epistemic uncertainty can be related to what the model does not yet know and can be reduced by observing more data for the sparse or unknown domain of the chemical space that the model has not fully learned [33]. A graphic illustration of these uncertainties is shown in Fig. 1.

Although separately quantifying aleatoric and epistemic uncertainties allows one to characterize the uncertainty sources [35], rationalizing the estimated uncertainty in the prediction through the chemical structure of the query molecule remains challenging. In practice, reasoning the prediction failure on a specific molecular structure is often done manually based on human intuition. Since predictions from black box models such as deep learning methods are challenging to interpret and analyze due to their non-transparency [36], Explainable Artificial Intelligence (XAI) has recently received much attention [37–39]. Explainability refers to the ability to explain why an artificial intelligence model has reached a particular decision or prediction [39]. Equipped with explainability that fits human intuition, the internal mechanisms of models become more understandable and trustworthy when applied to safety-critical tasks that demand careful decision-making [38]. For molecular property predictions, significant progress has been made to obtain a better understanding of model characteristics and behaviors by analyzing molecular graphs, compounds, atoms, or feature representations [40–42]. For the same reason, it is highly desirable to rationalize the estimated uncertainty through chemical structures to aid in understanding the reason behind the failure of the prediction (e.g., unrecognized functional groups or chemical structures that are rare in the dataset). Explaining the estimated uncertainty through molecular structures is also useful for determining out-of-domain chemicals and improving model performance through automatic selection of informative data, which are important research topics in active learning [14, 20] and drug discovery [17, 21, 43].

In this work, we develop an explainable uncertainty quantification method for the prediction of molecular properties based on deep learning. This method can separately quantify aleatoric and epistemic uncertainties and attribute these uncertainties to atoms in the molecule, which allows one to assess the reason behind the failure of a prediction. The atom-based uncertainty quantification method proposed in this work is adapted from the Deep Ensembles method [24], which has been used in many applications [12, 13, 44, 45]. However, similar to what Busk et al. observed [12], we found that Deep Ensembles can produce poorly calibrated aleatoric uncertainty estimations. To address this issue, we propose a *post-hoc* calibration method to refine the aleatoric uncertainty of Deep Ensembles. Unlike previous works that emphasize finding a scaling factor for calibrating the uncertainty of out-of-domain datasets [46–48], we focus on fine-tuning the weights of selected layers of ensemble models for better calibrated aleatoric uncertainty estimates.

In short, the main contributions are listed as follows.

1. We develop an atom-based uncertainty model that can attribute the uncertainty to the atoms present in a molecule, which results in a better understanding of the chemical insight of the model.
2. We propose a *post-hoc* calibration scheme to improve the aleatoric uncertainty calculated with Deep Ensembles for better uncertainty quantification.

## Methods

In this section, we first introduce how Deep Ensembles calculate aleatoric and epistemic uncertainties, and then discuss the *post-hoc* calibration method we propose to improve Deep Ensembles for better uncertainty quantification. Lastly, we introduce the architecture of the atom-based uncertainty model, the evaluation metrics, and the datasets used to benchmark the performance of the different uncertainty estimation models.

### Approximate uncertainty with Deep Ensembles

The concept of quantifying both aleatoric and epistemic uncertainty in one framework was presented by Kendall and Gal [31]. Meanwhile, the idea of applying the ensemble method to estimate the model uncertainty of deep learning models (Deep Ensembles) was first proposed by Lakshminarayanan et al. [24]. In practice, Deep Ensembles can be considered an alternative approximation to Bayesian inference [49] and can be implemented in two approaches: ensembling and bootstrapping [13, 24], both of which are based on assembling several networks trained independently. Ensembling trains multiple networks with different initial weights such that each loss reaches different local minima, so the prediction of a query may vary across the networks. The extent of discrepancy in the predictions reflects the epistemic uncertainty of the model. On the other hand, bootstrapping trains multiple networks by randomly sampling data from the dataset with replacement. With partially different training data, each network learns to predict a certain portion of the data in the original dataset. In this work, we apply Deep Ensembles with the ensembling approach as recommended by Lakshminarayanan et al. [24].

In this study, we assume the inherent noise in the data (aleatoric uncertainty) follows a Gaussian distribution [13]. Since Deep Ensembles combine the predictions of $M$ networks, the final predictive distribution is assumed as a uniformly-weighted mixture of Gaussian distributions [24]. We note that if the type of noise is known in advance, the output distribution does not need to be Gaussian and can be approximated with a function closer to the actual noise distribution [13, 24]. To predict a Gaussian distribution with a neural network, the last layer of the network can be modified into two parallel layers that output the mean ($\mu (x)$) and variance (${\sigma }^{2}(x)$) of the Gaussian function [50]. The objective of optimizing a set of distributions is to maximize the likelihood function of Gaussian. Given a dataset $\mathcal{D}=\{{x}_{k}, {y}_{k}{\}}_{k=1}^{N}$ where ${y}_{k}=\mu \left({x}_{k}\right)+\epsilon \left({x}_{k}\right)$ with $\epsilon \left({x}_{k}\right)\sim \mathcal{N}\left(0, {\sigma }^{2}\left({x}_{k}\right)\right)$[51], the target probability distribution for input ${x}_{k}$ can be written as

1 $$P\left( {{ }y_{k} {|}x_{k} } \right) = \left( {2\pi \sigma^{2} \left( {x_{k} } \right)} \right)^{{ - \frac{1}{2}}} \cdot exp\left( { - \frac{{(y_{k} - \mu \left( {x_{k} } \right))^{2} }}{{2\sigma^{2} \left( {x_{k} } \right)}}} \right)$$

where the $\mu ({x}_{k})$ is the mean and ${\sigma }^{2}\left({x}_{k}\right)$ is the variance [52].

Given a neural network model $m$ and assuming a predictive distribution $\widehat{{\varvec{y}}}$ consists of a mean ${\mu }_{m}({x}_{k})$ and a variance ${\sigma }_{m}^{2}({x}_{k})$ such that $\widehat{{\varvec{y}}}\sim \mathcal{N}({\mu }_{m}({x}_{k}), {\sigma }_{m}^{2}({x}_{k}))$, the optimal weights can be found by maximizing likelihood estimation (Eq. 1), which is equivalent to minimizing the negative log-likelihood (NLL), i.e., the heteroscedastic loss, of the predictive distributions.

2 $$- \ln \left( L \right) \propto \mathop \sum \limits_{k = 1}^{N} \frac{1}{{2\sigma_{m}^{2} \left( {x_{k} } \right)}}(y_{k} - \mu_{m} \left( {x_{k} } \right))^{2} { } + \frac{1}{2}\ln \left( {\sigma_{m}^{2} \left( {x_{k} } \right)} \right) + \frac{1}{2}\ln \left( {2\pi } \right)$$

An uncertainty model trained with the heteroscedastic loss minimizes NLL by tuning the predicted mean ${\mu }_{m}(x)$ and variance ${\sigma }_{m}^{2}(x)$ at the same time. Since aleatoric uncertainty is the noise in data, the output variance ${\sigma }_{m}^{2}({x}_{k})$ is defined as the aleatoric uncertainty of sample *k,* whose value depends on the absolute error between the true value ${y}_{k}$ and the mean ${\mu }_{m}({x}_{k})$ predicted by model $m$ (Eq. 2) [50]. The underlying assumption of this approach is that the error between ${y}_{k}$ and ${\mu }_{m}({x}_{k})$ is solely caused by the data noise in ${y}_{k}$. However, in practice, the function approximation for ${\mu }_{m}({x}_{k})$ may also contribute to the error, so the aleatoric uncertainty predicted by this method is model-dependent, and may be overestimated when the data is poorly-predicted by the model [53].

Because Deep Ensembles combine the predictions of $M$ models, the ensemble prediction is a mixture of Gaussian ${\widehat{{\varvec{y}}}}_{{\varvec{e}}{\varvec{n}}{\varvec{s}}}=\frac{1}{M}\sum_{m=1}^{M}\widehat{{\varvec{y}}}$ where the ensemble mean value ${\mu}_{ens}$ is calculated by averaging the output means of *M* models

3 $$\mu_{ens} = \frac{1}{M}\mathop \sum \limits_{m = 1}^{M} \mu_{m}$$

and the ensemble variance ${\sigma }_{ens}^{2}$ equals to

4 $$\sigma_{ens}^{2} = \frac{1}{M}\mathop \sum \limits_{m}^{M} \left( {\sigma_{m}^{2} + \mu_{m}^{2} } \right) - \mu_{ens}^{2} = \frac{1}{M}\mathop \sum \limits_{m = 1}^{M} \sigma_{m}^{2} + \frac{1}{M}\mathop \sum \limits_{m = 1}^{M} \left( {\mu_{m} - \mu_{ens} } \right)^{2}$$

where $\frac{1}{M}\sum_{m=1}^{M}{\sigma }_{m}^{2}$ and $\frac{1}{M}\sum_{m=1}^{M}{\left({\mu}_{m}-{\mu}_{ens}\right)}^{2}$ are the aleatoric and epistemic uncertainty of the ensemble prediction [12, 24, 31]

5 $$\sigma_{ale}^{2} = \frac{1}{M}\mathop \sum \limits_{m = 1}^{M} \sigma_{m}^{2} ,\,\, \sigma_{epi}^{2} = \frac{1}{M}\mathop \sum \limits_{m = 1}^{M} \left( {\mu_{m} - \mu_{ens} } \right)^{2}$$

### Post-hoc calibration for Deep Ensembles

Deep Ensembles proposed by Lakshminarayanan et al. is a simple and popular non-Bayesian approximation for modeling epistemic uncertainty. In Deep Ensembles, the aleatoric uncertainty is estimated by averaging the predicted variances from $M$ models (Eq. 5). Since each model is trained with the heteroscedastic loss function (Eq. 2), the aleatoric uncertainty predicted with model $m$ (${\sigma }_{m}^{2}$) should be well calibrated to represent the errors between true values and the mean calculated with model $m$ (${\mu }_{m}$). As the ensemble model estimates its mean value by averaging predicted means from $M$ models, the error of the ensemble model is expected to be reduced [12], which is theoretically accompanied by a lower aleatoric uncertainty compared with that of the individual model. However, averaging the prediction of aleatoric uncertainty made by each model (Eq. 5) does not generally reduce the magnitude of aleatoric uncertainty, which leads to an overestimation of ${\sigma }_{ale}^{2}$ and an underconfident ensemble model [12].

**Fig. 2:**
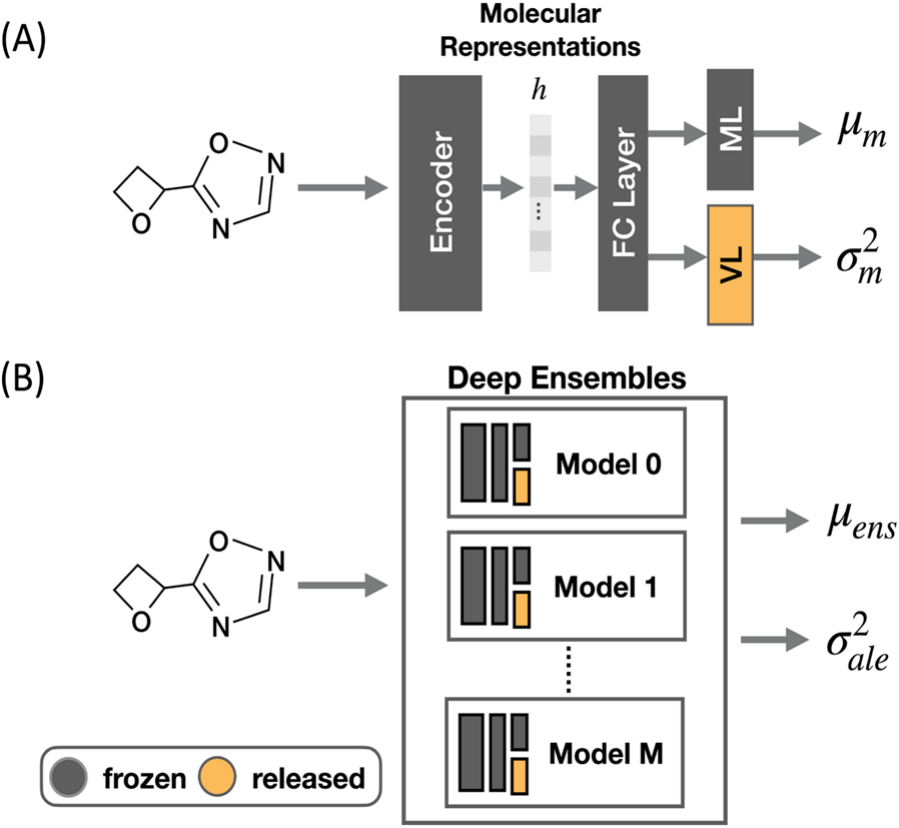
Distribution mean and variance predicted by (**A**) a neural network model and (**B**) Deep Ensembles. Because Deep Ensembles estimate the mean value ${\mu }_{ens}$ by averaging the predicted means of $M$ neural networks, the variance associated with Deep Ensembles (${\sigma }_{ale}^{2}$) is expected to be lower than the variance of one network in the ensemble (${\sigma }_{m}^{2}$). Therefore, one may overestimate ${\sigma }_{ale}^{2}$ by averaging ${\sigma }_{m}^{2}$ of networks in the ensemble (Eq. 5). This problem can be resolved by refining the weights in the variance layers (highlighted in yellow) to minimize the heteroscedastic loss function calculated with ${\mu }_{ens}$ and ${\sigma }_{ale}^{2}$ in the second round of training. FC, ML, and VL layers refer to the fully-connected layer, mean layer, and variance layer

To address this issue, we propose an intuitive *post-hoc* calibration method to improve the quality of aleatoric uncertainty of the ensemble model by retraining a portion of the weights of the networks. As shown in Fig. 2A, a neural network model with predicted uncertainty contains two output layers for predicting the mean and variance of a predictive distribution. In the *post-hoc* calibration, the networks in the ensemble are trained with relaxation of only the weights in variance layers (VL), keeping all the other weights frozen, so the calibration only affects the value of aleatoric uncertainty (Fig. 2B). In the calibration process, the mean and aleatoric uncertainty derived from the ensemble (${\mu }_{ens}$ and ${\sigma }_{ale}^{2}$) are used to calculate the heteroscedastic loss to ensure that ${\sigma }_{ale}^{2}$ can correctly represent the errors between the true value and the ${\mu }_{ens}$ predicted by the ensemble model. Note that since only the weights in variance layers are retrained during *post-hoc* training, the output mean ${\mu }_{m}$ in each model remains unchanged so the values of ${\mu }_{ens}$ and ${\sigma }_{epi}^{2}$ stay the same after the calibration procedure.

### Molecule- and atom-based uncertainty models

**Fig. 3:**
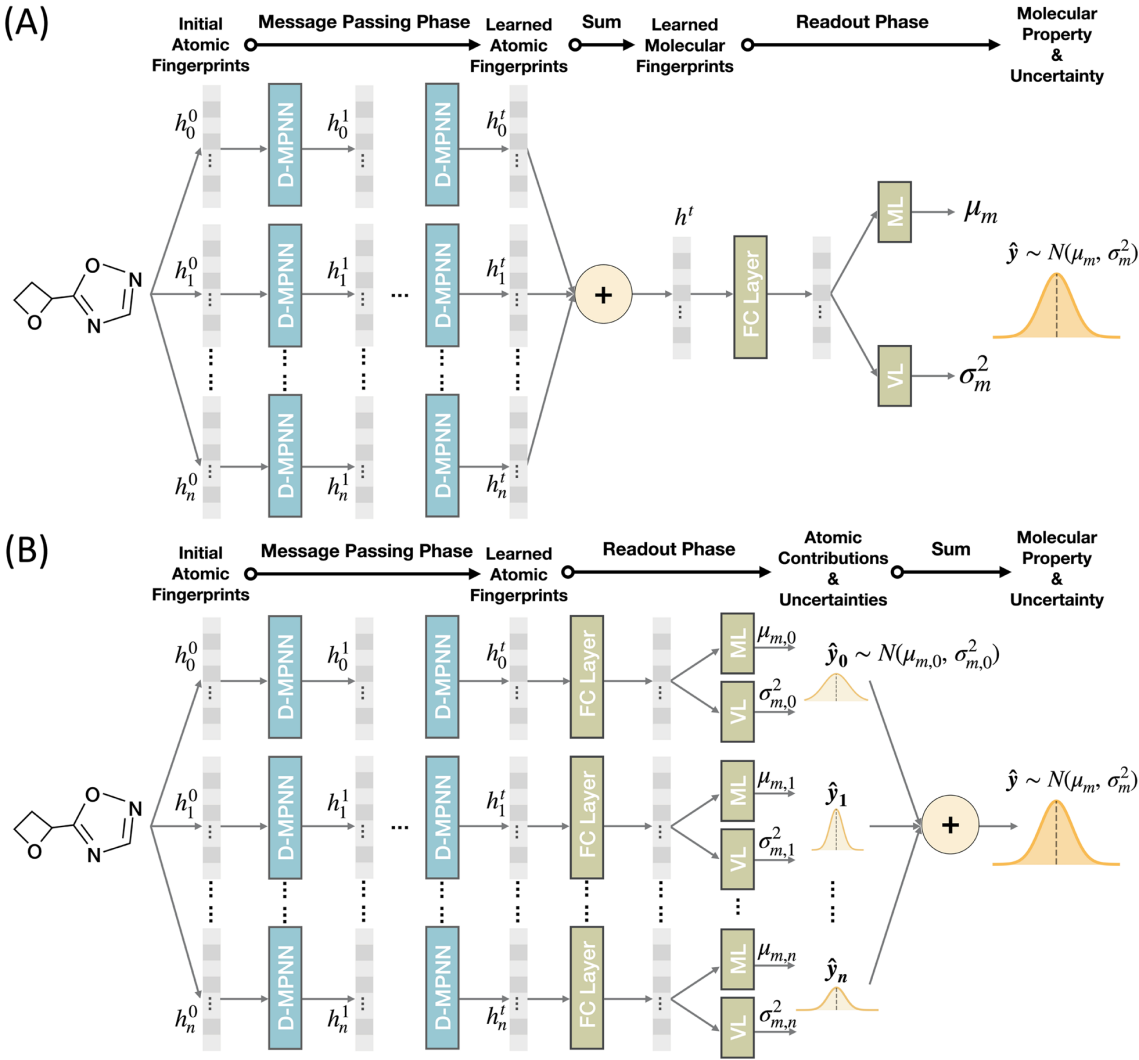
The architecture of the (**A**) molecule-based and (**B**) atom-based uncertainty model. The network takes molecular graphs with initial atoms and bonds information as input (${h}_{i}^{0}$). With $t$ iteration through D-MPNN message passing, each atom exchanges information with its neighbor atoms to generate the learned atomic fingerprints ${h}_{i}^{t}$. In (**A**), the learned atomic fingerprints are summed to form the learned molecular fingerprints. The molecular representation is passed into the fully-connected layer (FC Layer), and then into the mean layer (ML) and variance layer (VL), respectively, to obtain the mean ${\mu }_{m}$ and variance ${\sigma }_{m}^{2}$ of molecular property distribution $\widehat{y}$. On the other hand, in (**B**), the learned atomic fingerprints are passed into the FC Layer, ML, and VL to predict the property distribution ${\widehat{y}}_{i}$ of each atom separately. The molecular property distribution $\widehat{y}$ is obtained by aggregating ${\widehat{y}}_{i}$ of each atom in the molecule

Various schemes have been proposed to encode molecular structures into vector representations suitable for conventional machine learning algorithms [54]. In this work, we adopt the Directed Message Passing Neural Network (D-MPNN) [55], a 2D graph convolutional model, to encode molecular structures. This model contains message passing and readout phases as shown in Fig. 3. The implementation of the message passing phase in this work follows the Chemprop model [1], the details of which can be found in the work of Yang et al. [1]. In brief, the input is a graph including nodes (atoms) and edges (bonds) information of a molecule. The D-MPNN concatenates atom information with bond information linked with the atom to form initial fingerprints (${h}_{i}^{0}$). The atom and bond features contained in ${h}_{i}^{0}$ are summarized in Additional file 1: Tables S1 and S2, which were selected following the work of Chen et al. [56]. In the bond-level message passing procedure, each atom collects information from its neighbor atoms with bond direction considered and passes through layers and activation functions with $t$ iterations, resulting in atomic fingerprints with local, global, and directional knowledge (${h}_{i}^{t}$). In the original setting of Chemprop, these hidden vertex features are summed together to derive a molecular fingerprint, which is then passed to the next readout phase to predict molecular property and uncertainty (Fig. 3A) [1, 13].

In this work, we introduce the atom-based uncertainty method in which the learned atomic fingerprints are passed separately to the next readout phase to predict atom-based properties and uncertainties instead of pooled together to form the molecular fingerprint. As shown in Fig. 3B, we modified the readout phase of Chemprop to predict the atomic property contributions and the associated uncertainties, which are then aggregated to derive molecular property and molecular uncertainty. The algorithm at the readout phase is the main difference between our work and the molecule-based uncertainty model (Fig. 3A) proposed previously [1, 13]. In the atom-based uncertainty method, the molecular property distribution $\widehat{{\varvec{y}}}$ with mean ${\mu }_{m}$ and variance ${\sigma }_{m}^{2}$ is regarded as the sum of atomic Gaussian distributions ${\widehat{{\varvec{y}}}}_{{\varvec{i}}}$ of $n$ atoms in a molecule.

6 $${\widehat{\varvec{y}}} \sim N(\mu_{m} , \sigma_{m}^{2} ),\,\,{\widehat{\varvec{y}}_{\varvec{i}}} \sim N(\mu_{m,i} , \sigma_{m,i}^{2} )$$

7 $$\widehat{\varvec{y}} = \mathop \sum \limits_{{{i} = 1}}^{{n}} \widehat{\varvec{y}}_{{\varvec{i}}}$$

In detail, the atomic fingerprint of atom *i* derived from the message passing phase (${h}_{i}^{t}$) is passed into the fully-connected layers, which predict the atomic mean ${\mu }_{m,i}$ through the mean layer ${f}_{m}\left(\cdot \right)$ and atomic standard deviation ${\sigma }_{m,i}$ through the variance layer ${g}_{m}\left(\cdot \right)$. The mean of the molecular property ${\mu }_{m}$ is simply the summation of the atomic mean of each atom.

8 $$\mu_{m} = \mathop \sum \limits_{i = 1}^{n} \mu_{{m,i{ }}} = \sum_{i = 1}^{n} f_{m} \left( {h_{i}^{t} } \right)$$

On the other hand, the molecular variance ${\sigma }_{m}^{2}$ can be derived by summing the elements of a covariance matrix of which diagonal elements correspond to the atomic variance ${\sigma }_{m,i}^{2}$ and off-diagonal elements correspond to the covariance terms $cov({a}_{i}, {a}_{j})$ between each atom in the molecule [57]

9 $$\sigma_{m}^{2} = \mathop \sum \limits_{i = 1}^{n} \sigma_{{m,i{ }}}^{2} + { }2\mathop \sum \limits_{i = 2}^{n} \mathop \sum \limits_{j = 1}^{i - 1} cov\left( {\widehat{{{\varvec{y}}_{{\varvec{i}}} }},{ }\widehat{{{\varvec{y}}_{{\varvec{j}}} }}} \right)$$

10 $$\sigma_{m,i}=g_{m} \left( {h_{i}^{t} } \right),\,cov(\widehat{{\varvec{y}_{\varvec{i}} }},\widehat{{\varvec{y}_{\varvec{j}} }}) = \rho_{ij} \cdot \sigma_{i} \cdot \sigma_{j}$$

where ${\sigma }_{i}$ and ${\sigma }_{j}$ are the standard deviation of $\widehat{{{\varvec{y}}}_{{\varvec{i}}}}$ and $\widehat{{{\varvec{y}}}_{{\varvec{j}}}}$, and ${\rho }_{ij}$ is the correlation coefficient between atoms $i$ and $j$. In this work, we use the Pearson correlation coefficient [58] of the learned atomic fingerprints ${h}_{i}^{t}$ and ${h}_{j}^{t}$ to estimate the correlation between the property values of atom *i* and *j*

11 $$\rho_{ij} = \rho (\widehat{{\varvec{y}_{\varvec{i}} }},\widehat{{\varvec{y}_{\varvec{j}} }}) = \rho_{m} (h_{i}^{t} ,h_{j}^{t} ) \cong \,\frac{{\sum {_{v = 1}^{d} \left( {h_{i,v}^{t} - \overline{{h_{i}^{t} }} } \right)\,\left( {h_{j,v}^{t} - \overline{{h_{j}^{t} }} } \right)} }}{{\sqrt {\sum {_{v=1}^{d} \left( {h_{i,v}^{t} - \overline{h_{i}^{t}} } \right)^{2} \, } } \sqrt {\sum {_{v=1}^{d} \left( h_{j,v}^{t} - \overline{h_{j}^{t} } \right)^{2} } \,}}}$$

where ${h}_{i,v }^{t}$ is the $v$ ^th^ element of the fingerprint ${h}_{i}^{t}$. Similar to the molecule-based uncertainty method, one can aggregate the outputs of a number of atom-based uncertainty models to derive an ensemble mean and variance following the procedures discussed in the above subsection (Eq. 3–5).

### Evaluation metrics

We use mean absolute error (MAE) and root mean square error (RMSE) as the evaluation metrics of property prediction accuracy

12 $$MAE = \frac{1}{N}\left( {\mathop \sum \limits_{k = 1}^{N} \left| {\mu_{ens} \left( {x_{k} } \right){ } - y_{k} } \right|} \right)$$

13 $$RMSE = \sqrt {\frac{1}{N}\left( {\mathop \sum \limits_{k = 1}^{N} \left( {\mu_{ens} \left( {x_{k} } \right){ } - y_{k} } \right)^{2} } \right)}$$

where $N$ is the number of samples for evaluation. Since there is no ground truth of uncertainties, evaluating predicted uncertainty with traditional benchmarks is difficult. In this work, we use the expected calibration error (ECE) and expected normalized calibration error (ENCE) as the evaluation metrics of predicted uncertainties [13, 30]. The details of these two metrics are discussed below.

***Confidence-based Calibration Curve and ECE ***The outputs of the uncertainty model are assumed to be the mean and variance of a Gaussian distribution. In principle, one can use the percentage of the samples whose true values fall within the confidence interval defined by the predictive distribution to evaluate the quality of the predicted variance. For a well-calibrated case, the probability that ${y}_{k}$ will fall within the confidence interval should equal the percentage of the confidence level. The confidence-based calibration curve examines the fraction of data that actually falls in each confidence level. The difference between the confidence level (e.g., 60% confidence level) and the empirical fraction (e.g., 57% of data fall within the confidence interval) is defined as ECE [13, 45]

14 $$ECE = \frac{1}{B}\left( {\mathop \sum \limits_{b = 1}^{B} \left| {CL_{b} - EF_{b} } \right|} \right)$$

where $B$ is the number of confidence levels considered, $C{L}_{b}$ is the percentage of confidence level $b$, and $E{F}_{b}$ is the fraction of data points falling within confidence interval $b$*.*

***Error-based Calibration Curve and ENCE ***The error-based calibration curve examines the consistency between the expected error (measured by mean squared error, MSE) and the predicted uncertainty ${\sigma }^{2}(x)$ under the assumption that the estimator is unbiased [59].

15 $$\forall \sigma , {\mkern 1mu} {\mathbb{E}}\left[ {\left( {\mu \left( x \right) - y} \right)^{2} | \sigma^{2} \left( x \right) = \sigma^{2} } \right] = \sigma^{2}$$

In practice, the testing data is sorted by the predicted uncertainty and divided into $B$ bins with $K$ data in each bin. The error-based calibration curve is a parity plot between the RMSE (Eq. 13) and the root mean uncertainty (RMU) of the data in each bin

16 $$RMU = \sqrt {\frac{1}{K}\mathop \sum \limits_{k = 1}^{K} \sigma_{k}^{2} }\,\,.$$

The difference between the expected error (RMU) and error of prediction (RMSE) is what ENCE calculates, and a lower ENCE means a better calibration

17 $$ENCE = \frac{1}{B}\mathop \sum \limits_{b}^{B} \frac{{\left| {RMSE_{b} - RMU_{b} } \right|}}{{RMU_{b} }}\,\,.$$

### Computational details

The datasets used for benchmarks in this work include QM9 [60], Zinc15 [61], Delaney [62], and Lipophilicity [63] (Table 1), which were accessed from MoleculeNet [64]. The molecule-based uncertainty model proposed by Scalia et al. [13] (Fig. 3A) was taken as the base model to validate the applicability of the *post-hoc* calibration method and the performance of the atom-based uncertainty quantification method. In this work, each ensemble model contains 30 networks. We apply the heteroscedastic loss (Eq. 2) during training to acquire aleatoric uncertainty and Deep Ensembles for epistemic uncertainty. As shown in Fig. 3, we use 2 D-MPNN layers to encode input molecules and 2 fully-connected layers, where the last layer contains two parallel layers outputting the mean and variance of the predictive distribution. Each dataset is randomly split into training, validation, and testing data in a ratio of 8:1:1. The early stopping was set to halt training if heteroscedastic loss of the validation data fails to decrease for more than 15 epochs.

**Table 1:** Summary of the Benchmark Datasets

Dataset	Property	Size
QM9	enthalpy $[\mathrm{kcal}\bullet {\mathrm{mol}}^{-1}]$	133,885
Zinc15	water/octanol partition coefficient $[\mathrm{logP}]$	250,000
Lipophilicity	water/octanol distribution coefficient at pH7.4 $[\mathrm{logD }7.4]$	4187
Delaney (ESOL)	water solubility $[\mathrm{log}(\mathrm{mol}\bullet {\mathrm{L}}^{-1})]$	1128

## Results and discussion

This section is organized as follows. We first present how the *post-hoc* calibration scheme improves the quality of aleatoric uncertainty of the ensemble model. Next, we compare the prediction accuracy and uncertainty performance between the molecule- and atom-based uncertainty models, and discuss how the atom-based uncertainty model can help to identify the chemical structures that lead to the failure of a prediction.

### Post-hoc calibration of aleatoric uncertainty

**Fig. 4:**
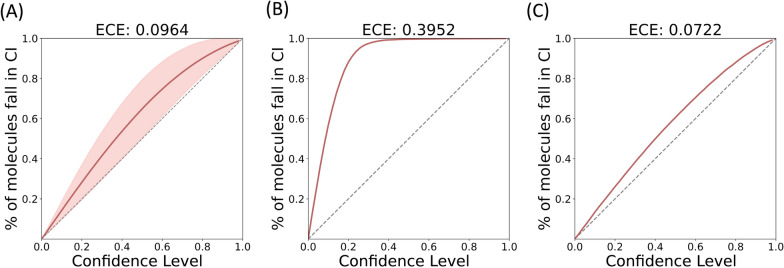
Confidence-based calibration curves and ECEs based on the aleatoric uncertainty for the Zinc15 testing set. The aleatoric uncertainty is calculated with (**A**) a single atom-based uncertainty model, (**B**) an ensemble of atom-based uncertainty models, and (**C**) an ensemble of atom-based uncertainty models after *post-hoc* calibration. The calibration procedure reduces the ECE of the ensemble method from 0.3952 to 0.0722. The shaded area shown in (**A**) is 95% CI calculated with 30 independent models

**Fig. 5:**
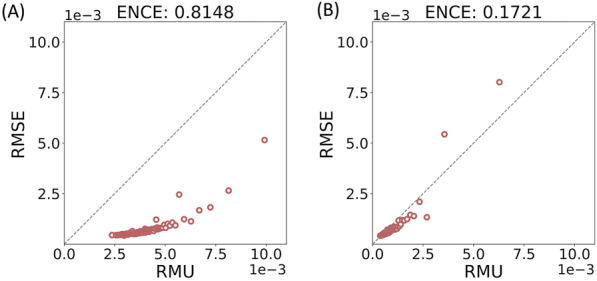
Error-based calibration curves and ENCEs based on the aleatoric uncertainty for the Zinc15 testing set. The aleatoric uncertainty is calculated with (**A**) an ensemble of atom-based uncertainty models and (**B**) an ensemble of atom-based uncertainty models after *post-hoc* calibration. The calibration procedure reduces the ENCE of the ensemble method from 0.8148 to 0.1721

The *post-hoc* calibration scheme aims to fine-tune the aleatoric uncertainty overestimated by the ensemble scheme. Since each network in the ensemble was trained to minimize its own heteroscedastic loss (Eq. 2), the calibration curve based on the aleatoric uncertainty of each network is close to the diagonal line (perfect calibration), which results in a low ECE as shown in Fig. 4A. However, because the error of the ensemble model is often lower than that of the individual model, simply averaging the aleatoric uncertainty of each individual model (Eq. 5) may overestimate ${\sigma }_{ale}^{2}$ of the ensemble model. Therefore, as shown in Fig. 4B and Fig. 5A, the confidence-based and error-based calibration curves for deep ensembles are far from perfect calibration, leading to higher ECE and ENCE than the single model. This problem can be alleviated using the *post-hoc* calibration procedure shown in Fig. 2B, which retrains the variance layer to output a lower and more calibrated uncertainty for the ensemble scheme (Figs. 4C and 5B). See Supporting Information for more discussions of how aleatoric uncertainty varies before and after *post-hoc* calibration (Additional file 1: Fig. S8).

Table 2 summarizes the ECE and ENCE values calculated with the atom-based and molecule-based uncertainty models for different chemical datasets before and after the *post-hoc* calibration. The confidence- and error-based calibration curves for these datasets can be found in the Supporting Information (Additional file 1: Figs. S1–S15). For most of the datasets we examined, the ECE and ENCE decrease after calibration, suggesting the quality of aleatoric uncertainty is generally improved through the calibration procedure. We note that the effectiveness of the *post-hoc* calibration depends on the error reduction that the ensemble model can achieve relative to the individual models it aggregates. When ensembling greatly reduces the error, the predicted aleatoric uncertainty of the ensemble model is largely overestimated by averaging ${\sigma }_{m}^{2}$ of individual models, and therefore the effect of the *post-hoc* calibration is pronounced. However, there are also cases in which ensembling does not significantly improve model performance. For example, the ensemble model does not outperform single models for the Lipophilicity dataset, so the predicted aleatoric uncertainty is not significantly overestimated before calibration (Table 2). In this case, the room for improving aleatoric uncertainty is very limited.

**Table 2:** ECE and ENCE performance of ensemble models before and after post-hoc calibration for different datasets

Dataset	Uncertainty model	Aleatoric uncertainty			
		ECE		ENCE	
		Before calibration	After calibration	Before calibration	After calibration
QM9	AtomUnc	0.1635	0.0129	0.3094	0.2120
	MolUnc	0.1270	0.0700	0.2507	0.1772
Zinc15	AtomUnc	0.3952	0.0722	0.8148	0.1721
	MolUnc	0.3950	0.3139	0.7415	0.6200
Lipophilicity	AtomUnc	0.0413	0.0396	0.3683	0.3704
	MolUnc	0.0157	0.0119	0.2452	0.2441
Delaney (ESOL)	AtomUnc	0.2118	0.0622	0.6414	0.5578
	MolUnc	0.1994	0.1121	0.6122	0.5468

AtomUnc and MolUnc refer to the atom- and molecule-based uncertainty models, respectively

### Comparison of atom- and molecule-based uncertainty models

The performance of property and uncertainty prediction of the atom- and molecule-based uncertainty models are listed in Table 3. For most of the testing sets, the MAE, RMSE, ECE, and ENCE of the atom-based uncertainty model are comparable to the molecule-based uncertainty model [13] proposed previously, which validates the usefulness of the atom-based architecture (Fig. 3B) in molecular property and uncertainty predictions. The advantage of the atom-based uncertainty model is that it provides an extra layer of chemical insight to the predicted uncertainty. Taking a molecular graph as input, the atom-based uncertainty model outputs not only the molecular property, but also the atomic contributions to the property and the associated uncertainties. With these outputs, one can better understand how the model attributes the property prediction and uncertainty to the atoms in the molecule, and therefore can quickly assess the reason behind the potential failure of a prediction. Examples to illustrate this point are given in the following subsection.

**Table 3:** Prediction accuracy and uncertainty performance of the atom-based uncertainty model (AtomUnc) and the molecule-based uncertainty model (MolUnc)

Dataset	Model	MAE	RMSE	ECE		ENCE	
				Ale	Epi	Ale	Epi
QM9	AtomUnc	0.8904	2.7023	0.0129	0.0311	0.2120	0.4724
	MolUnc	0.8394	2.7196	0.0700	0.0328	0.1772	0.5913
Zinc15	AtomUnc	0.00088	0.0083	0.0722	0.3284	0.1721	0.7238
	MolUnc	0.00123	0.0113	0.3139	0.3201	0.6200	0.7051
Lipophilicity	AtomUnc	0.4191	0.5952	0.0396	0.2294	0.3704	1.5324
	MolUnc	0.6709	0.8418	0.0119	0.3096	0.2441	1.9964
Delaney	AtomUnc	0.5031	0.6715	0.0622	0.0740	0.5578	0.6762
	MolUnc	0.5900	0.7520	0.1121	0.0203	0.5468	0.5314

ECE and ENCE are calculated based on aleatoric (Ale) and epistemic (Epi) uncertainties

### Analysis of atomic uncertainty

Because the atom-based uncertainty model attributes the predicted uncertainty to the atoms in a molecule, it can help to identify the chemical structures under-represented in the dataset and identify the types of species whose data potentially contain significant noise. To illustrate this point, we carried out two experiments with modified QM9 datasets to mimic scenarios in which data quality and quantity vary for different types of species. In the first experiment, artificial noises are added to data of nitrogen-containing molecules of QM9 to examine the capacity of the atom-based uncertainty model to capture the origin of data noise. On the other hand, in the second experiment, nitrogen-containing species are removed from QM9 to verify the ability of the atom-based uncertainty model to identify under-represented chemical structures. The results of these two experiments are discussed below.

***Heterogeneous data quality*** To verify that the predicted atomic aleatoric uncertainty can recognize the source of noise in a molecule, we created a noisy dataset ${\mathcal{D}}^{noise}=\{{x}_{k}, {y}_{k}^{noise}{\}}_{k=1}^{N}$ by adding $r$ independent Gaussian noises (mean $=$ 0, variance $=$ 1) to the molecules containing nitrogen atoms

18 $$y_{k}^{noise} = y_{k} + \sum\limits_{j = 1}^{r} { \epsilon_{j} }$$

where ${\epsilon }_{j}\sim \mathcal{N}(0, 1)$, ${y}_{k}$ is the true property value, and $r$ is the number of nitrogen atoms in molecule *k*. Note that the property of molecules without nitrogen atoms remains unchanged. A nitrogen-noisy model was trained with ${\mathcal{D}}^{noise}$, and a base model was trained with the unmodified dataset $\mathcal{D}=\{{x}_{k}, {y}_{k}{\}}_{k=1}^{N}$ for the purpose of comparison.

**Fig. 6:**
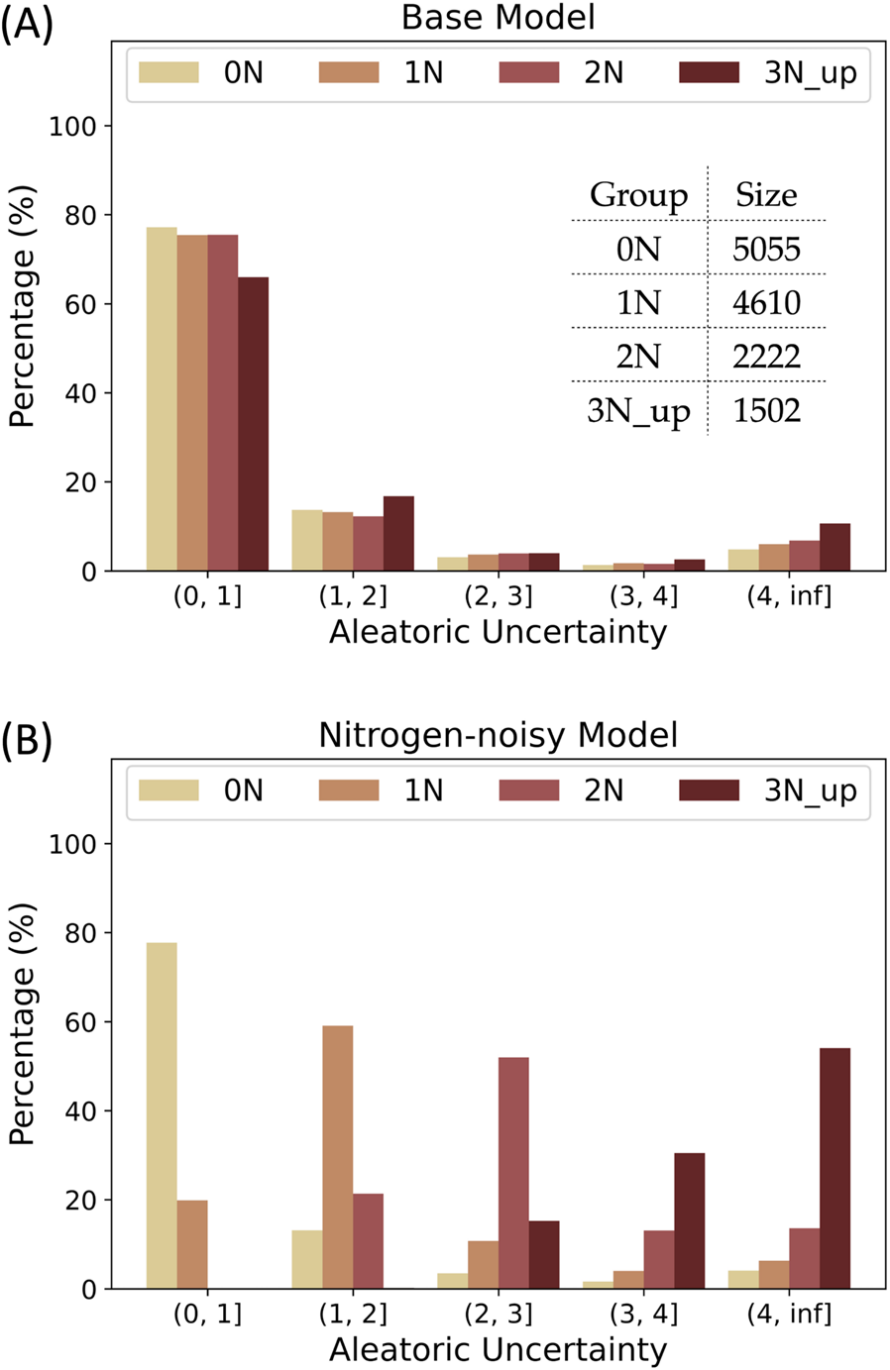
Aleatoric uncertainty distributions of QM9 testing data calculated with (**A**) the base model and (**B**) the nitrogen-noisy model. The testing data are grouped by the number of nitrogen atoms in the molecule. The molecules without nitrogen atoms are denoted as 0 N, molecules containing one nitrogen atom are denoted as 1 N, and so on

**Fig. 7:**
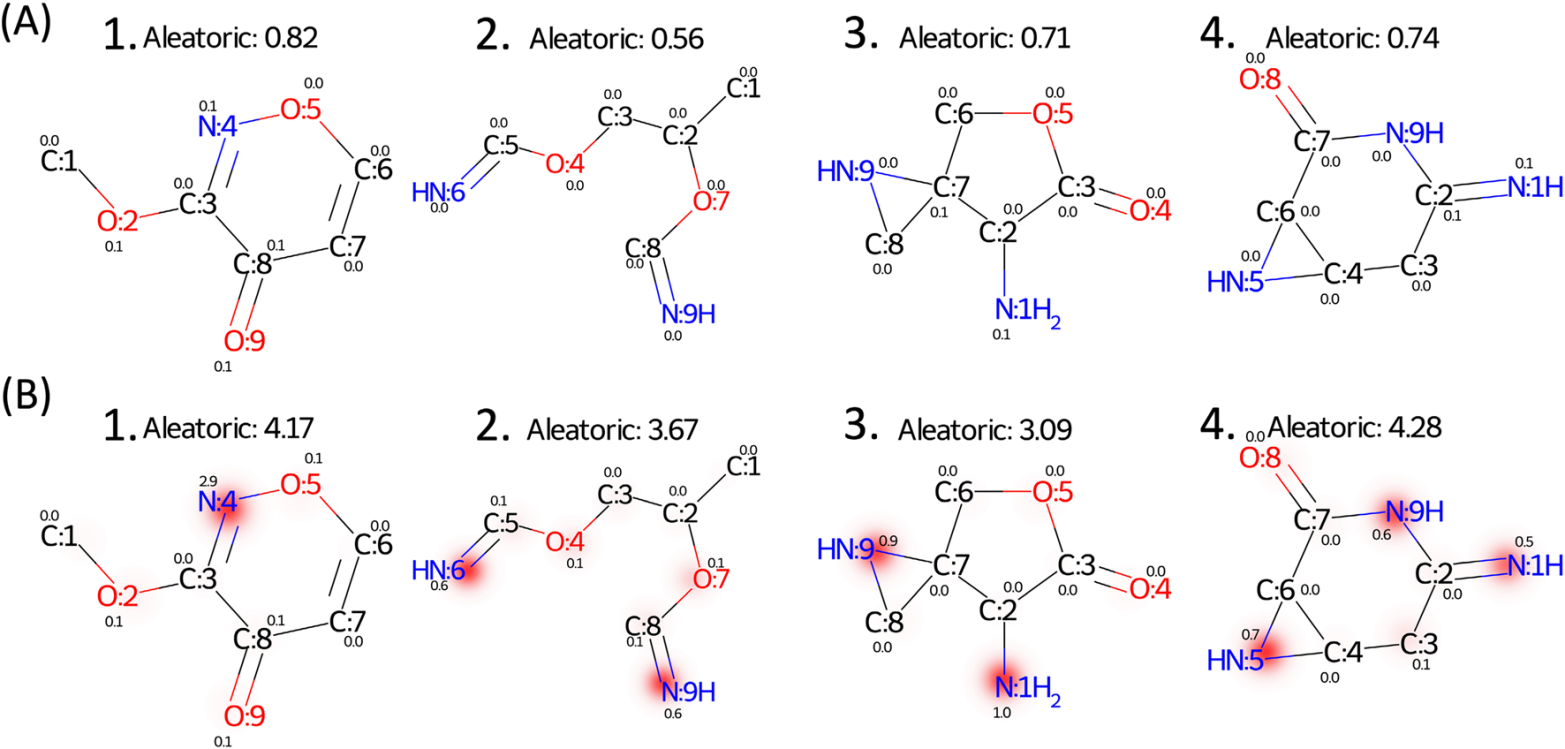
Aleatoric uncertainties of molecules with nitrogen atoms predicted by (**A**) the base model and (**B**) the nitrogen-noisy model. Numbers labeled at each atom are the predicted atomic aleatoric uncertainty

Aleatoric uncertainties of the testing data calculated with the nitrogen-noisy and base models are shown in Fig. 6. In the base model, most of the molecules have low predicted aleatoric uncertainties. Conversely, the distribution of aleatoric uncertainty shifts right (increases) as the number of nitrogen atoms in the molecules increases in the nitrogen-noisy model, which suggests that the model can successfully learn the artificial noise introduced in ${\mathcal{D}}^{noise}$. Figure 7 shows four test molecules with their molecular and atomic aleatoric uncertainties. Because these molecules contain nitrogen atoms, the molecular uncertainties predicted with the nitrogen-noisy model (Fig. 7B) are higher than those of the base model (Fig. 7A). Through the analysis of the atomic aleatoric uncertainty, one can see that the increase in uncertainty is concentrated at the nitrogen atoms.

***Heterogeneous data quantity*** Epistemic uncertainty indicates how unfamiliar the model is to a molecule. To examine whether the atomic epistemic uncertainty can detect unseen chemical structures, we removed the nitrogen-containing molecules from the QM9 dataset to train a nitrogen-ignorant model, and then compared it with the base model trained with the original QM9 dataset.

**Fig. 8:**
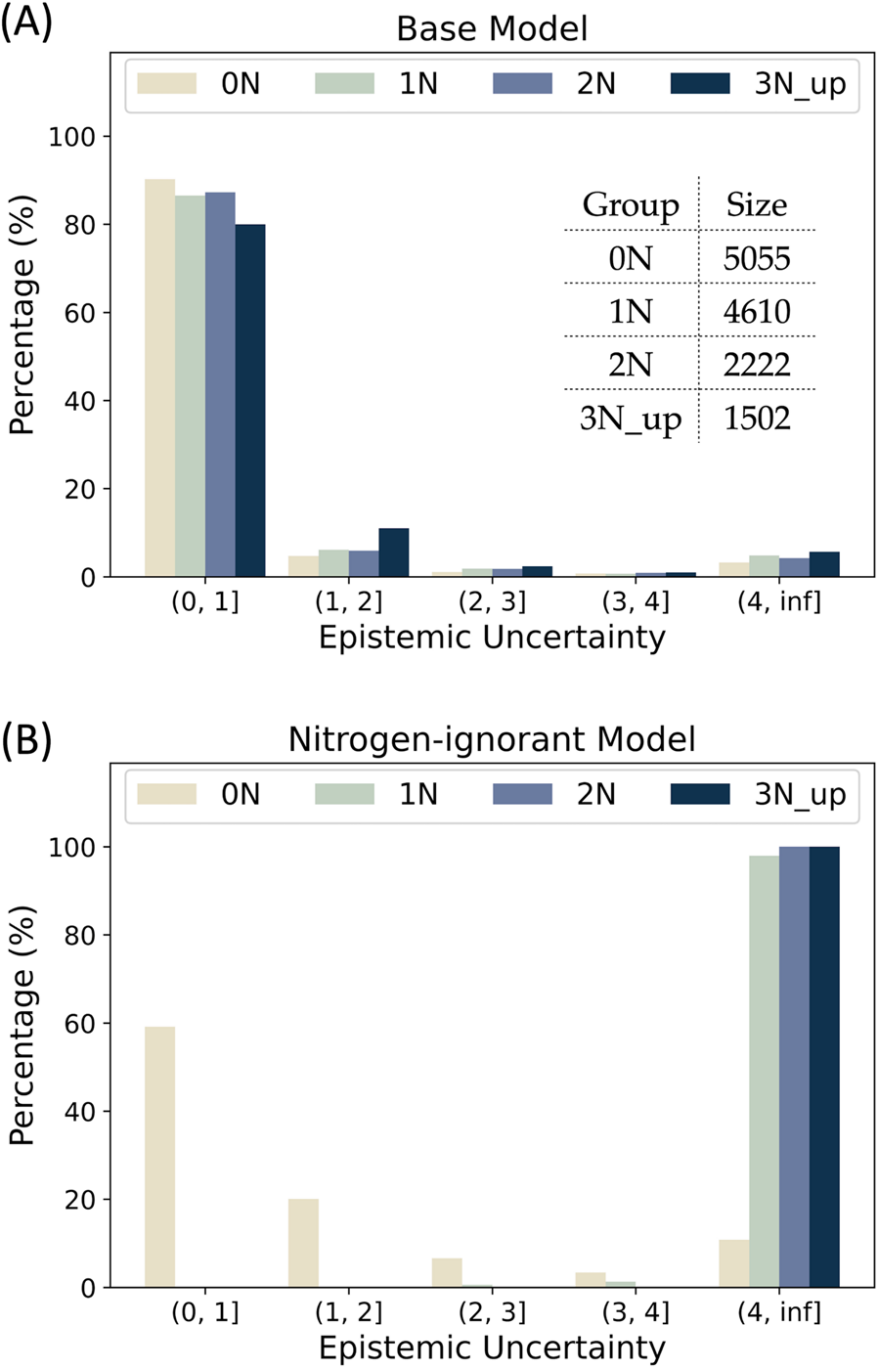
Epistemic uncertainty distributions of QM9 testing data calculated with (**A**) the base model and (**B**) the nitrogen-ignorant model. The testing data are grouped by the number of nitrogen atoms in the molecule. The molecules without nitrogen atoms are denoted as 0 N, molecules containing one nitrogen atom are denoted as 1 N, and so on

**Fig. 9:**
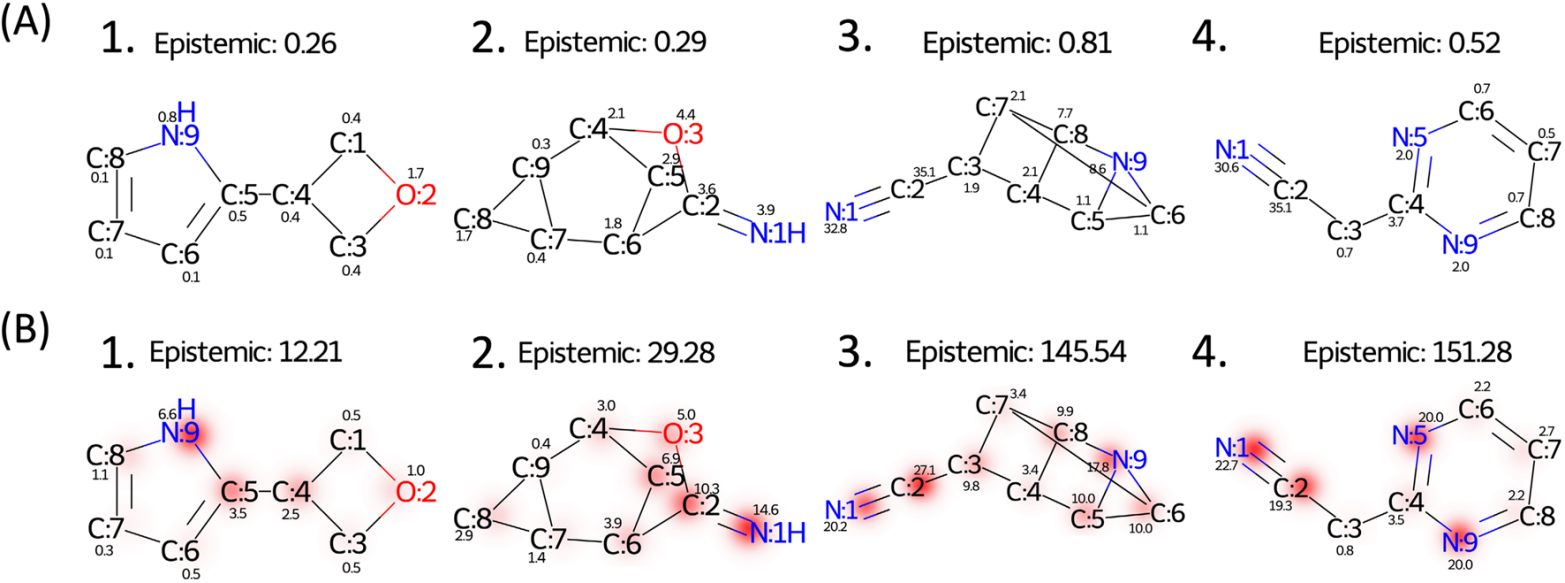
Epistemic uncertainties of molecules with nitrogen atoms predicted by (**A**) the base model and (**B**) the nitrogen-ignorant model. Numbers labeled at each atom are the predicted atomic epistemic uncertainty

Figure 8 shows the epistemic uncertainty of the test data predicted by the nitrogen-ignorant model and the base model. Because the base model has seen all types of molecules in the original dataset, most of the epistemic uncertainties of the test molecules predicted by the base model are low. On the other hand, because the nitrogen-ignorant model has not seen nitrogen-containing species, the epistemic uncertainties greatly increase for the nitrogen-containing molecules, which indicates the self-awareness of ignorance of an unseen domain. We note that because the sizes of the training and validation datasets decrease for the nitrogen-ignorant model, the overall error and uncertainty of the nitrogen-ignorant model are larger than the base model, even for molecules containing no nitrogen atoms (group 0 N in Fig. 8). Four test molecules with their atomic epistemic uncertainties are shown in Fig. 9. The nitrogen-ignorant model assigns relatively higher atomic epistemic uncertainty to the nitrogen atoms, indicating that the atom-based model is capable of identifying the unseen chemical structure.

The experiments discussed above show that the model estimates a higher aleatoric uncertainty for the species whose data are associated with significant noise and a larger epistemic uncertainty for the species that are under-represented. However, we note that when one uses Deep Ensembles, a high estimate of aleatoric uncertainty is not always caused by data noise. For instance, when we removed the nitrogen-containing molecules from the QM9 dataset, we observed an increase in the estimate of aleatoric uncertainty for the nitrogen-containing species in the test set (Additional file 1: Fig. S18). This is because the weights associated with the nitrogen atom were not trained, so the network outputs for nitrogen-containing species (including the estimate of aleatoric uncertainty) were significantly mispredicted. Similarly, in Deep Ensembles, a high estimate of epistemic uncertainty is not always caused by a lack of data. For example, when there was significant noise in the data, finding the optimal fit became more challenging, which might also result in a larger discrepancy in the predictions of Deep Ensembles, and hence accidentally lead to an overestimation of epistemic uncertainty (Additional file 1: Fig. S17). Therefore, the uncertainty derived from Deep Ensembles should be interpreted with care, and further method improvement may be required.

We note that molecules with low molecular uncertainty can sometimes contain atoms with large atomic uncertainty. For example, some of the atomic uncertainties shown in Fig. 9A are larger than the value of the molecular uncertainty. In the atom-based model, the molecular property is calculated as the sum of atomic property values (Eq. 7), so the variance of the molecular property is equal to the sum of the total variances of each atom and the covariances between all possible pairs of atoms. Since the covariances between atoms may be negative, the molecular property variance can be lower than the variance of each atom. This situation mainly occurs when there are multiple ways to distribute contribution value to each atom, which results in low confidence in the atomic property but high confidence in the molecule-level prediction. More discussions on this point can be found in the Supporting Information (Additional file 1: Fig. S16).

## Conclusions

In this study, we propose an atom-based uncertainty quantification method for deep learning-based molecular property prediction. This atom-based model can learn the property contributions of atoms and the associated aleatoric and epistemic uncertainties. Our experiments suggest that the atomic aleatoric uncertainty can help to identify the types of species whose data are potentially associated with significant noises, and the atomic epistemic uncertainty can help to determine the chemical structure with which the model is unfamiliar. Given the explainability and transparency of the model, one can be aware not only of the potential failure of a prediction, but also of the reasons why the prediction may fail through its atomic uncertainties. Moreover, we introduce a *post-hoc* calibration method to fine-tune the overestimated aleatoric uncertainty of ensemble models. The improved quality of aleatoric uncertainty is indicated through the reduction of ECE and ENCE for a wide range of molecular property prediction tasks.

## Supplementary Information

**Additional file 1.** Additional information as noted in the text, including confidence- and error-based calibration curves for the datasets listed in Table 2, distributions of confidence intervals, correlation coefficient matrixes for atomic uncertainties, complete lists of atom and bond features, computational costs required to train the uncertainty model, and more analysis of epistemic and aleatoric uncertainty, are provided. Additional figures S1–S18 and additional tables S1–S4.

## Data Availability

The source code of the atom-based uncertainty model can be found in our GitHub repository: https://github.com/chuiyang/atom-based_uncertainty_model. The molecule-based uncertainty model developed by Scalia et al. is also available on GitHub: https://github.com/gscalia/chemprop/tree/uncertainty. The datasets used in this study are obtained from MoleculeNet (https://moleculenet.org/).

## Abbreviations

DNN: Deep neural networks
BNN: Bayesian neural networks
NLL: Negative log-likelihood
ML: Mean layer
VL: Variance layer
FC: Fully-connected
D-MPNN: Directed message passing neural network
MAE: Mean absolute error
RMSE: Root mean square error
ECE: Expected calibration error
ENCE: Expected normalized calibration error
RMU: Root mean uncertainty
CI: Confidence interval

## References

[1] Yang K, Swanson K, Jin W (2019). Analyzing learned molecular representations for property prediction. J Chem Inf Model.

[2] Chithrananda S, Grand G, Ramsundar B (2020) ChemBERTa: Large-scale self-supervised pretraining for molecular property prediction. arXiv. 10.48550/arXiv.2010.09885

[3] Chen H, Engkvist O, Wang Y (2018). The rise of deep learning in drug discovery | Elsevier enhanced reader. Drug Discov Today.

[4] Angermueller C, Pärnamaa T, Parts L, Stegle O (2016). Deep learning for computational biology. Mol Syst Biol.

[5] Segler MHS, Waller MP (2017). Neural-symbolic machine learning for retrosynthesis and reaction prediction. Chem A Eur J.

[6] Schreck JS, Coley CW, Bishop KJM (2019). Learning retrosynthetic planning through simulated experience. ACS Cent Sci.

[7] Meuwly M (2021). Machine learning for chemical reactions. Chem Rev.

[8] Cai L, Zhu Y (2015). The challenges of data quality and data quality assessment in the big data era. Data Sci J.

[9] Rodrigues T (2019). The good, the bad, and the ugly in chemical and biological data for machine learning. Drug Discov Today Technol.

[10] Ovadia Y, Fertig E, Ren J et al (2019) Can you trust your model’s uncertainty? evaluating predictive uncertainty under dataset shift. arXiv. 10.48550/arXiv.1906.02530

[11] Nigam A, Pollice R, Hurley MFD (2021). Assigning confidence to molecular property prediction. Expert Opin Drug Discov.

[12] Busk J, Jørgensen PB, Bhowmik A (2021). Calibrated uncertainty for molecular property prediction using ensembles of message passing neural networks. Mach Learn Sci Technol.

[13] Scalia G, Grambow CA, Pernici B (2020). Evaluating scalable uncertainty estimation methods for deep learning-based molecular property prediction. J Chem Inf Model.

[14] Hao Z, Lu C, Huang Z (2020). ASGN: an active semi-supervised graph neural network for molecular property prediction.

[15] Musil F, Willatt MJ, Langovoy MA, Ceriotti M (2019). Fast and accurate uncertainty estimation in chemical machine learning. J Chem Theory Comput.

[16] Lamb G, Paige B (2020) Bayesian graph neural networks for molecular property prediction. arXiv. 10.48550/arXiv.2012.02089

[17] Soleimany AP, Amini A, Goldman S (2021). Evidential deep learning for guided molecular property prediction and discovery. ACS Cent Sci.

[18] Kosasih EE, Cabezas J, Sumba X et al (2021) On graph neural network ensembles for large-scale molecular property prediction. arXiv. 10.48550/arXiv.2106.15529

[19] Imbalzano G, Zhuang Y, Kapil V (2021). Uncertainty estimation for molecular dynamics and sampling. J Chem Phys.

[20] Li Y-P, Han K, Grambow CA, Green WH (2019). Self-evolving machine: a continuously improving model for molecular thermochemistry. J Phys Chem A.

[21] Gubaev K, Podryabinkin EV, Shapeev AV (2018). Machine learning of molecular properties: locality and active learning. J Chem Phys.

[22] Wang H, Yeung D-Y (2016). Towards bayesian deep learning: a framework and some existing methods. IEEE Trans Knowl Data Eng.

[23] Kucukelbir A, Tran D, Ranganath R, et al (2017) Automatic differentiation variational inference. J Mach Learn Res 18:430–474

[24] Lakshminarayanan B, Pritzel A, Blundell C (2016) Simple and scalable predictive uncertainty estimation using deep ensembles. arXiv. 10.48550/arXiv.1612.01474

[25] Gal Y, Ghahramani Z (2016) Dropout as a bayesian approximation: representing model uncertainty in deep learning. PMLR, pp 1050–1059

[26] Blundell C, Cornebise J, Kavukcuoglu K, Wierstra D (2015) Weight uncertainty in neural network. In: proceedings of the 32nd international conference on machine learning. PMLR, pp 1613–1622

[27] Alaa A, Schaar MVD (2020) Discriminative Jackknife: quantifying uncertainty in deep learning via higher-order influence functions. In: proceedings of the 37th international conference on machine learning. PMLR, pp 165–174

[28] Lin Z, Trivedi S, Sun J (2021) Locally valid and discriminative confidence intervals for deep learning models. https://arxiv.org/abs/2106.00225

[29] Romano Y, Patterson E, Candes E (2019) Conformalized quantile regression. In: advances in neural information processing systems. curran associates, Inc

[30] Hirschfeld L, Swanson K, Yang K (2020). Uncertainty quantification using neural networks for molecular property prediction. J Chem Inf Model.

[31] Kendall A, Gal Y (2017) What uncertainties do we need in bayesian deep learning for computer vision? arXiv. 10.48550/arXiv.1703.04977

[32] Hüllermeier E, Waegeman W (2021). Aleatoric and epistemic uncertainty in machine learning: an introduction to concepts and methods. Mach Learn.

[33] Kiureghian AD, Ditlevsen O (2009). Aleatory or epistemic? Does it matter?. Struct Saf.

[34] Griffiths R-R, Aldrick AA, Garcia-Ortegon M (2022). Achieving robustness to aleatoric uncertainty with heteroscedastic Bayesian optimisation. Mach Learn Sci Technol.

[35] Kwon Y, Won J-H, Kim BJ, Paik MC (2020). Uncertainty quantification using Bayesian neural networks in classification: application to biomedical image segmentation. Comput Stat Data Anal.

[36] Koh PW, Liang P (2017) Understanding black-box predictions via influence functions. PMLR, pp 1885–1894

[37] Xu F, Uszkoreit H, Du Y, Tang J, Kan M-Y, Zhao D (2019). Explainable AI: a brief survey on history, research areas, approaches and challenges. Natural language processing and chinese computing.

[38] Linardatos P, Papastefanopoulos V, Kotsiantis S (2021). Explainable AI: a review of machine learning interpretability methods. Entropy.

[39] Barredo Arrieta A, Díaz-Rodríguez N, Del Ser J, et al (2020) Explainable Artificial Intelligence (XAI): Concepts, taxonomies, opportunities and challenges toward responsible AI. Information Fusion 58:82–115. 10.1016/j.inffus.2019.12.012

[40] Rodríguez-Pérez R, Bajorath J (2021). Explainable machine learning for property predictions in compound optimization: miniperspective. J Med Chem.

[41] Rao J, Zheng S, Yang Y (2021). Quantitative evaluation of explainable graph neural networks for molecular property prediction. Patterns.

[42] Jiménez-Luna J, Grisoni F, Schneider G (2020). Drug discovery with explainable artificial intelligence. Nat Mach Intell.

[43] Eyke SN, Green HW, Jensen FK (2020). Iterative experimental design based on active machine learning reduces the experimental burden associated with reaction screening. React Chem Eng.

[44] Weigert M, Schmidt U, Boothe T (2018). Content-aware image restoration: pushing the limits of fluorescence microscopy. Nat Methods.

[45] Gustafsson FK, Danelljan M, Schön TB (2020) Evaluating scalable bayesian deep learning methods for robust computer vision. arXiv:190601620

[46] Kuleshov V, Fenner N, Ermon S (2018) Accurate uncertainties for deep learning using calibrated regression. In: Proceedings of the 35th International Conference on Machine Learning. PMLR, pp 2796–2804

[47] Laves M-H, Ihler S, Fast JF, et al (2021) Recalibration of aleatoric and epistemic regression uncertainty in medical imaging. arXiv:210412376

[48] Guo C, Pleiss G, Sun Y, Weinberger KQ (2017) On calibration of modern neural networks. PMLR 1321–1330

[49] Bernardo JM, Smith AFM (2009). Bayesian theory.

[50] Nix DA, Weigend AS (1994) Estimating the mean and variance of the target probability distribution. In: proceedings of 1994 IEEE international conference on neural networks (ICNN’94). pp 55–60 vol.1

[51] Cawley GC, Talbot NLC, Foxall RJ (2004). Heteroscedastic kernel ridge regression. Neurocomputing.

[52] Cawley GC, Talbot NLC, Chapelle O, Quiñonero-Candela J, Dagan I, Magnini B, d’Alché-Buc F (2006). Estimating predictive variances with kernel ridge regression. Machine Learning Challenges Evaluating Predictive Uncertainty, Visual Object Classification, and Recognising Tectual Entailment.

[53] Seitzer M, Tavakoli A, Antic D, Martius G (2022) On the pitfalls of heteroscedastic uncertainty estimation with probabilistic neural networks

[54] Wigh DS, Goodman JM, Lapkin AA (2022). A review of molecular representation in the age of machine learning. WIREs Computational Mol Sci.

[55] Dai H, Dai B, Song L (2016) Discriminative embeddings of latent variable models for structured data. In: proceedings of the 33rd international conference on machine learning. PMLR, pp 2702–2711

[56] Chen L-Y, Hsu T-W, Hsiung T-C, Li Y-P (2022) Deep Learning-Based Increment Theory for Formation Enthalpy Predictions. J Phys Chem A 126:7548–7556. 10.1021/acs.jpca.2c04848 10.1021/acs.jpca.2c04848 36217924

[57] Bertsekas DP, Tsitsiklis JN (2008). Introduction to probability.

[58] Benesty J, Chen J, Huang Y, Cohen I, Cohen Israel, Huang Yiteng, Chen Jingdong, Benesty Jacob (2009). Pearson Correlation Coefficient Noise Reduction in Speech Processing. Noise reduction in speech processing.

[59] Levi D, Gispan L, Giladi N, Fetaya E (2020) Evaluating and calibrating uncertainty prediction in regression tasks. arXiv:190511659 10.3390/s22155540 PMC9330317 35898047

[60] Ramakrishnan R, Dral PO, Rupp M, von Lilienfeld OA (2014). Quantum chemistry structures and properties of 134 kilo molecules. Sci Data.

[61] Sterling T, Irwin JJ (2015). ZINC 15—ligand discovery for everyone. J Chem Inf Model.

[62] Delaney JS (2004). ESOL: estimating aqueous solubility directly from molecular structure. J Chem Inf Comput Sci.

[63] Mendez D, Gaulton A, Bento AP (2019). ChEMBL: towards direct deposition of bioassay data. Nucleic Acids Res.

[64] Wu Z, Ramsundar B, Feinberg EN (2018). MoleculeNet: a benchmark for molecular machine learning. Chem Sci.

