# Supplementary material for: Explainable uncertainty quantifications for deep learning-based molecular property prediction
Source: J Cheminform. 2023 Feb 3;15:13. doi: 10.1186/s13321-023-00682-3 (PMC9898940; doi:10.1186/s13321-023-00682-3)
Supplement: Supplementary file 1 — Additional file 1. Additional information as noted in the text, including confidence- and error-based calibration curves for the datasets listed in Table 2, distributions of confidence intervals, correlation coefficient matrixes for atomic uncertainties, complete lists of atom and bond features, computational costs required to train the uncertainty model, and more analysis of epistemic and aleatoric uncertainty, are provided. Additional figures S1–S18 and additional tables S1–S4. [file 13321_2023_682_MOESM1_ESM.docx]

**Explainable Uncertainty Quantifications for Deep Learning-Based Molecular Property Prediction**

Chu-I Yang^[a]^ and Yi-Pei Li^*[a][b]^

[a] Department of Chemical Engineering, National Taiwan University, No. 1, Sec. 4, Roosevelt Road, Taipei 10617, Taiwan.

[b] Taiwan International Graduate Program (TIGP), Academia Sinica, No. 128, Sec. 2, Academia Road, Taipei 11529, Taiwan.

*E-mail: yipeili@ntu.edu.tw

Supporting Information

**Confidence-based Calibration Curve**

Figure 4 in the manuscript shows the confidence-based calibration curve for Zinc15 based on the aleatoric uncertainty predicted by the atom-based uncertainty model. The confidence-based calibration curves for other datasets and those predicted by the molecule-based uncertainty model are shown below. The ECEs of these calibration curves are summarized in Table 2 in the manuscript.


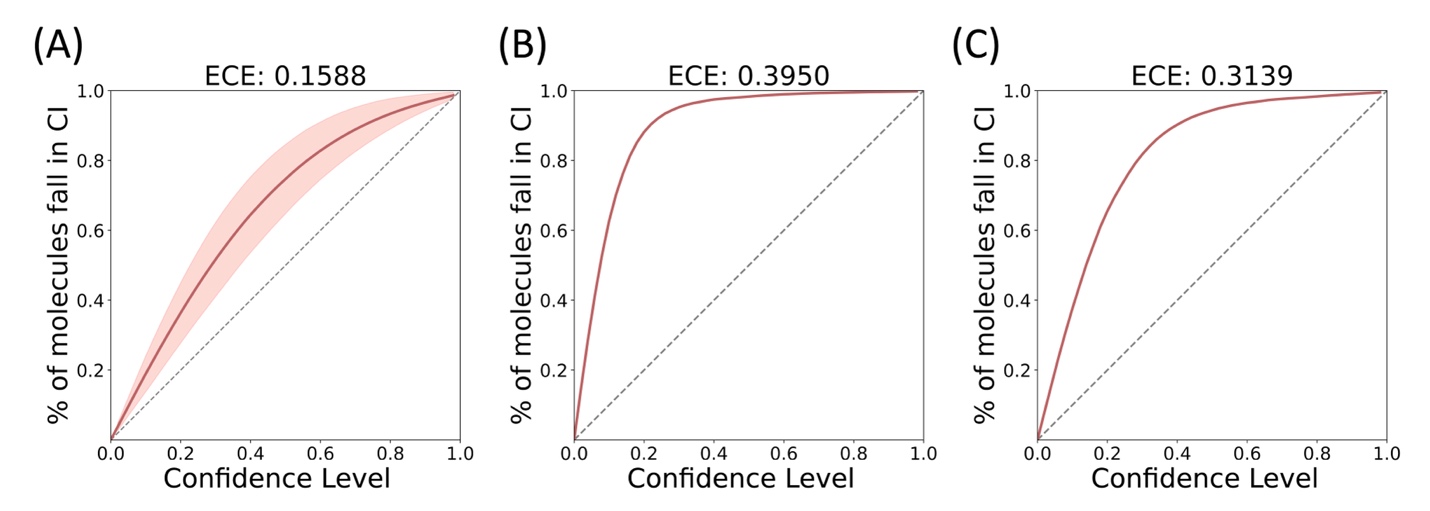


**Figure S1.** Confidence-based calibration curves and ECEs for the Zinc15 testing set based on the aleatoric uncertainty calculated with (A) a single molecule-based uncertainty model, (B) an ensemble of molecule-based uncertainty models, and (C) an ensemble of molecule-based uncertainty models after *post-hoc* calibration. The shaded area shown in (A) is 95% CI calculated with 30 independent models.


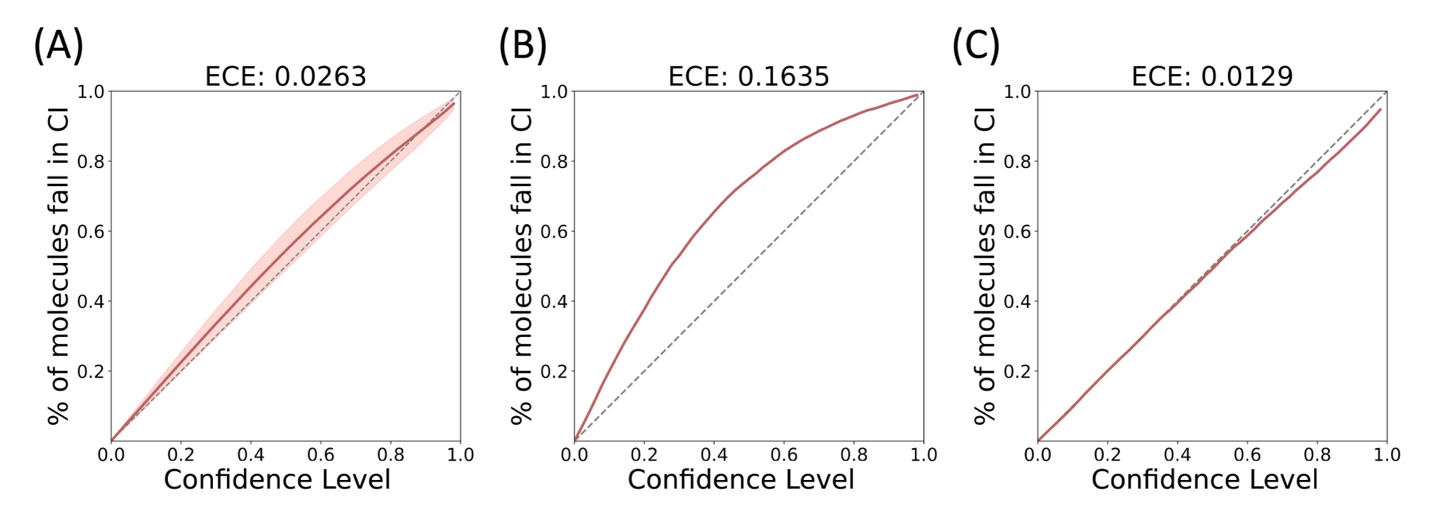


**Figure S2.** Confidence-based calibration curves and ECEs for the QM9 testing set based on the aleatoric uncertainty calculated with (A) a single atom-based uncertainty model, (B) an ensemble of atom-based uncertainty models, and (C) an ensemble of atom-based uncertainty models after *post-hoc* calibration. The shaded area shown in (A) is 95% CI calculated with 30 independent models.


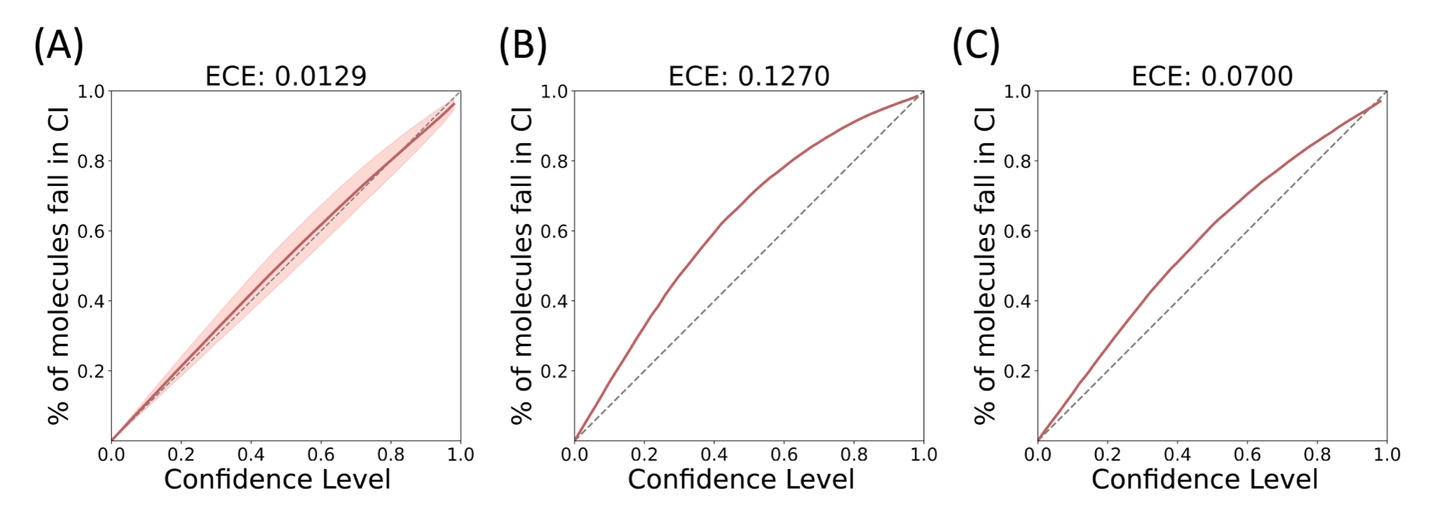


**Figure S3.** Confidence-based calibration curves and ECEs for the QM9 testing set based on the aleatoric uncertainty calculated with (A) a single molecule-based uncertainty model, (B) an ensemble of molecule-based uncertainty models, and (C) an ensemble of molecule-based uncertainty models after *post-hoc* calibration. The shaded area shown in (A) is 95% CI calculated with 30 independent models.


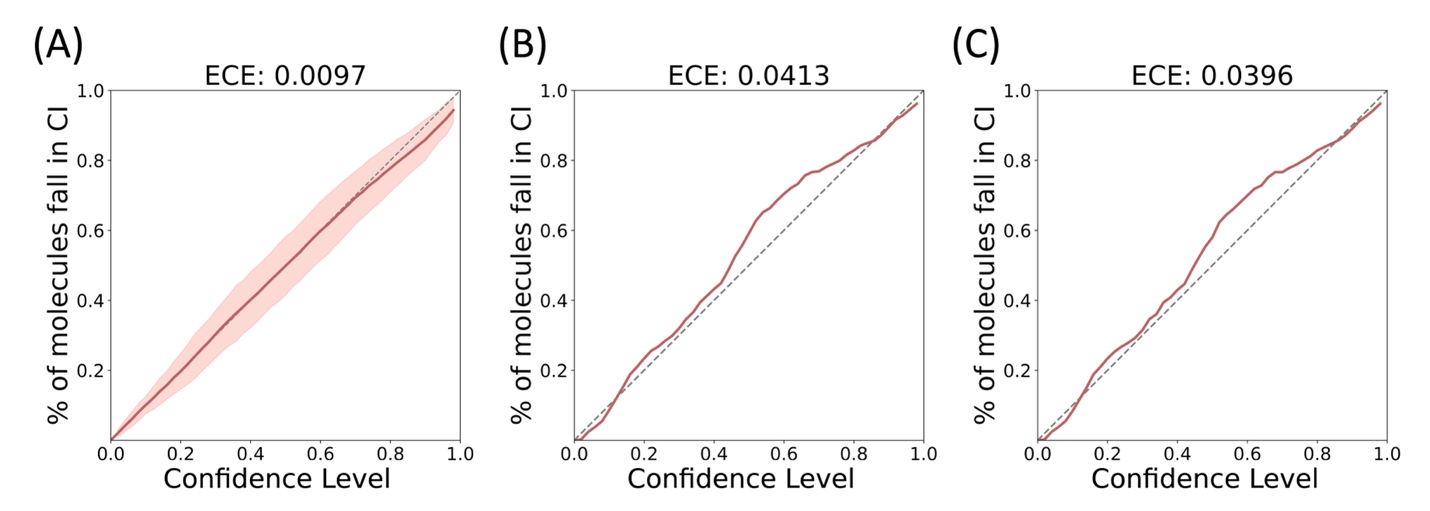


**Figure S4.** Confidence-based calibration curves and ECEs for the Lipophilicity testing set based on the aleatoric uncertainty calculated with (A) a single atom-based uncertainty model, (B) an ensemble of atom-based uncertainty models, and (C) an ensemble of atom-based uncertainty models after *post-hoc* calibration. The shaded area shown in (A) is 95% CI calculated with 30 independent models.


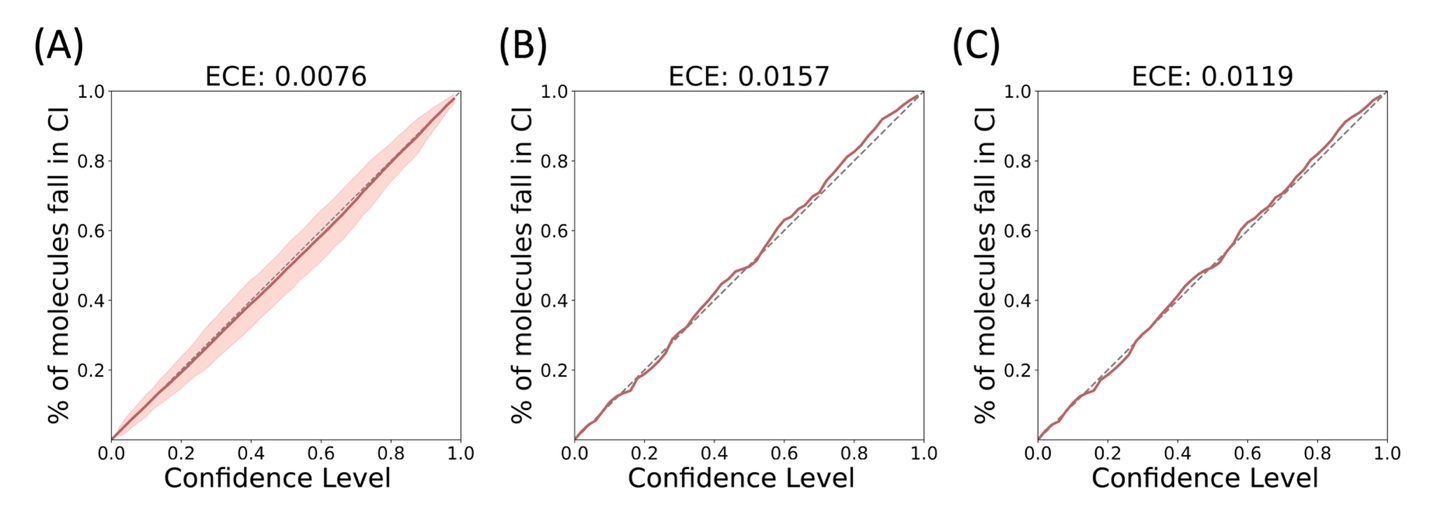


**Figure S5.** Confidence-based calibration curves and ECEs for the Lipophilicity testing set based on the aleatoric uncertainty calculated with (A) a single molecule-based uncertainty model, (B) an ensemble of molecule-based uncertainty models, and (C) an ensemble of molecule-based uncertainty models after *post-hoc* calibration. The shaded area shown in (A) is 95% CI calculated with 30 independent models.


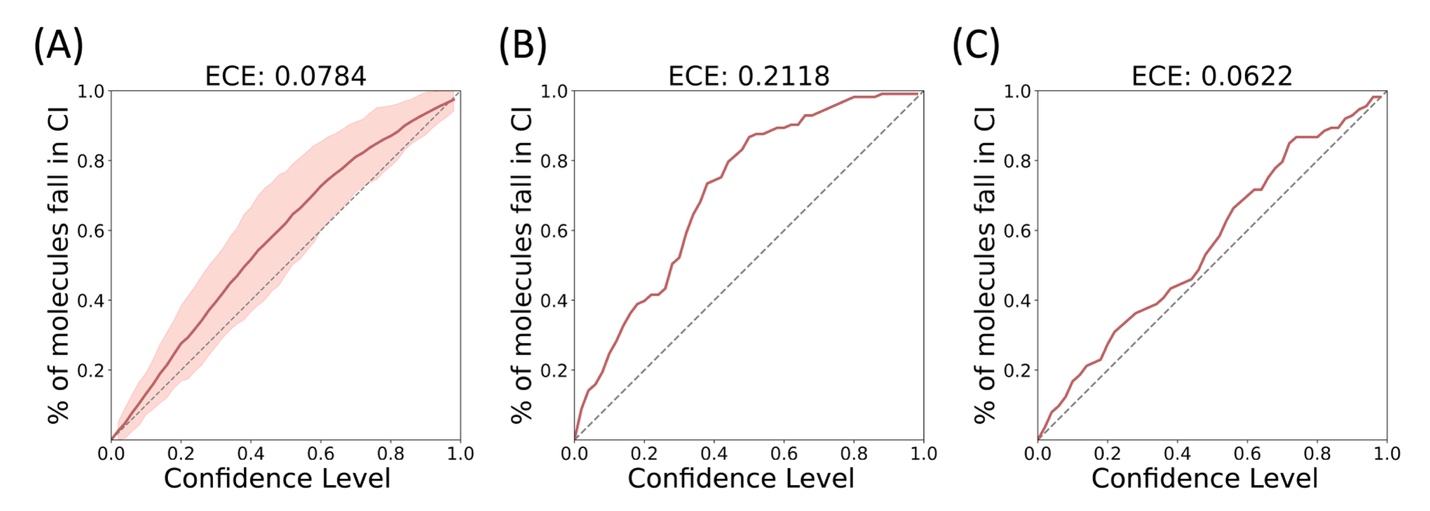


**Figure S6.** Confidence-based calibration curves and ECEs for the Delaney testing set based on the aleatoric uncertainty calculated with (A) a single atom-based uncertainty model, (B) an ensemble of atom-based uncertainty models, and (C) an ensemble of atom-based uncertainty models after *post-hoc* calibration. The shaded area shown in (A) is 95% CI calculated with 30 independent models.


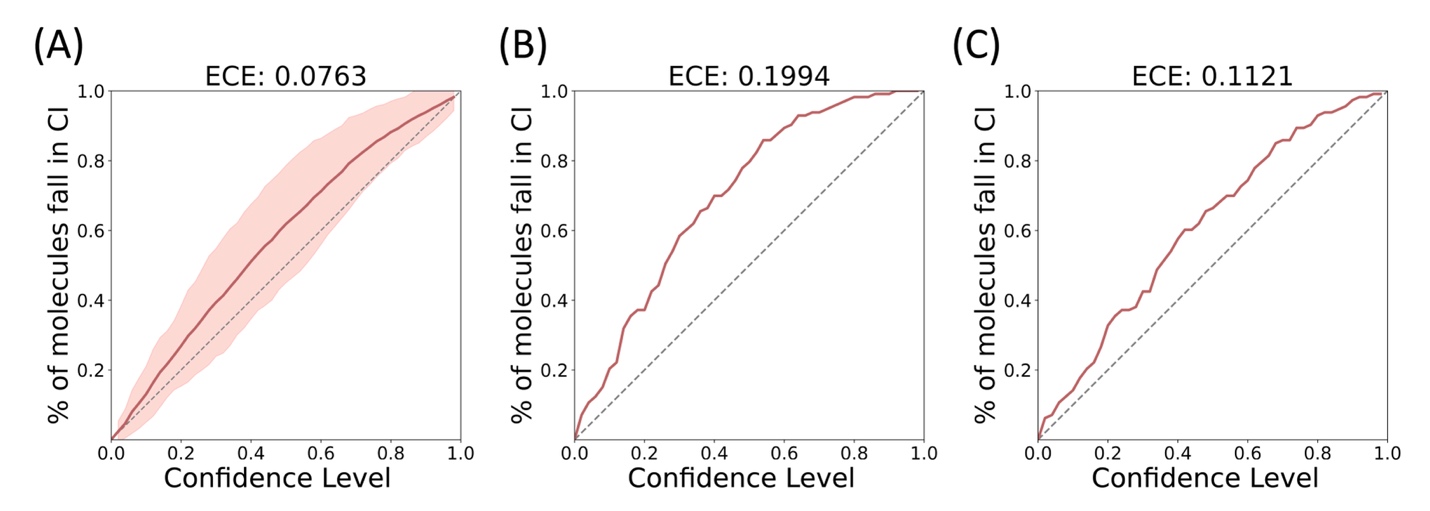


**Figure S7.** Confidence-based calibration curves and ECEs for the Delaney testing set based on the aleatoric uncertainty calculated with (A) a single molecule-based uncertainty model, (B) an ensemble of molecule-based uncertainty models, and (C) an ensemble of molecule-based uncertainty models after *post-hoc* calibration. The shaded area shown in (A) is 95% CI calculated with 30 independent models.

**Distributions of Confidence Intervals**

Figure S8 shows the distribution of the confidence interval widths of the testing data of Zinc 15 (25000 samples in the set) before and after *post-hoc* calibration. The distribution shifts left after calibration, suggesting the predicted aleatoric uncertainties generally decrease through the calibration procedure. This finding suggests that the model becomes more confident and hence can better represent the confidence intervals of the ensemble model, as the error of the ensemble model is expected to be reduced.


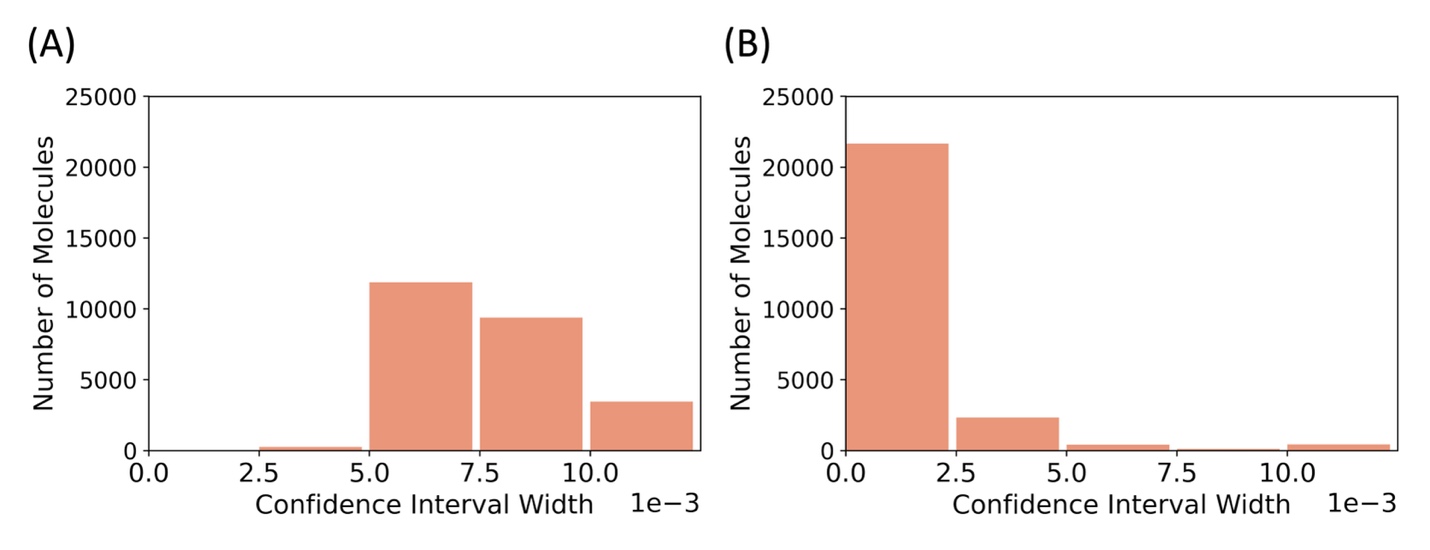


**Figure S8.** The distribution of the 63.8% confidence interval of the test molecules of Zinc 15 (A) before and (B) after *post-hoc* calibration.

**Error-based Calibration Curve**

Figure 5 in the manuscript shows the error-based calibration curve for Zinc15 based on the aleatoric uncertainty predicted by the atom-based uncertainty model. The error-based calibration curves for other datasets and those predicted by the molecule-based uncertainty model are shown below. The ENCEs of these calibration curves are summarized in Table 2 in the manuscript.


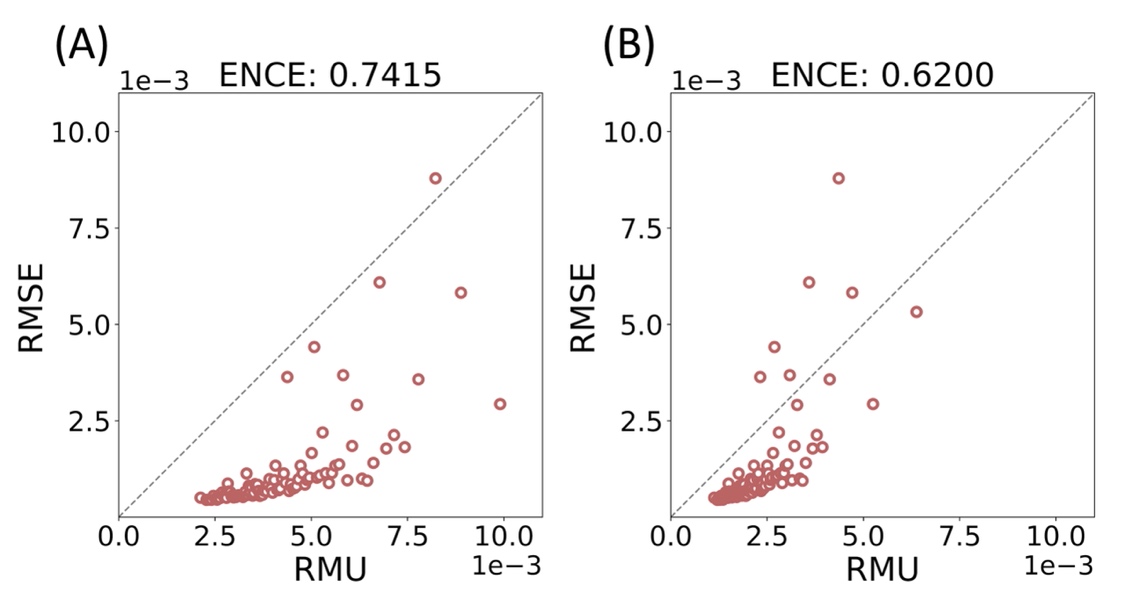


**Figure S9.** Error-based calibration curves and ENCEs for the Zinc15 testing set based on the aleatoric uncertainty calculated with (A) an ensemble of molecule-based uncertainty models and (B) an ensemble of molecule-based uncertainty models after *post-hoc* calibration.


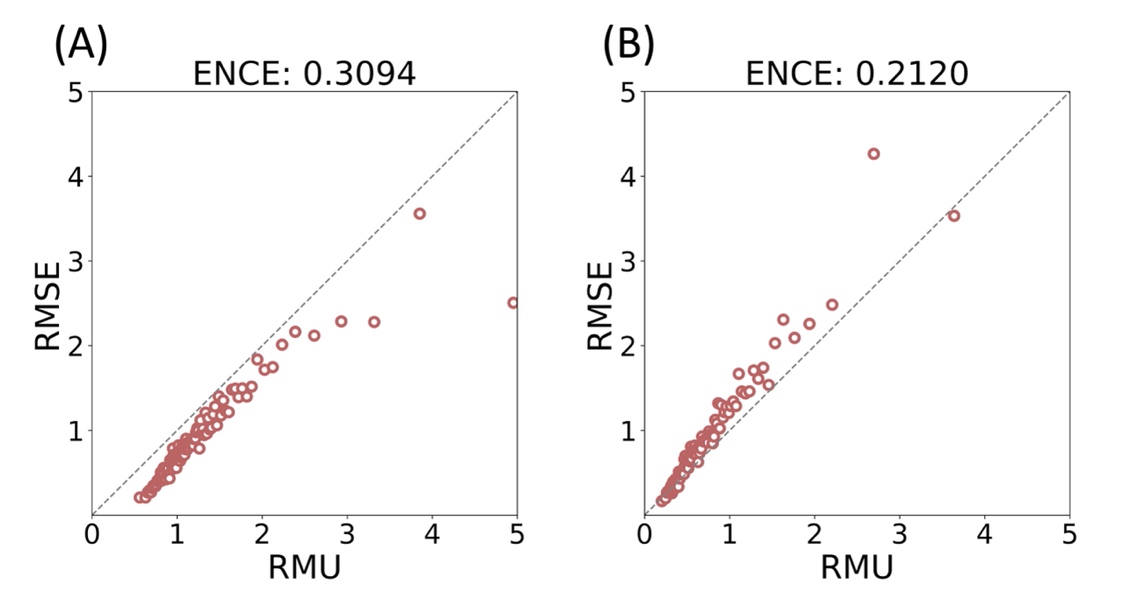


**Figure S10.** Error-based calibration curves and ENCEs for the QM9 testing set based on the aleatoric uncertainty calculated with (A) an ensemble of atom-based uncertainty models and (B) an ensemble of atom-based uncertainty models after *post-hoc* calibration.


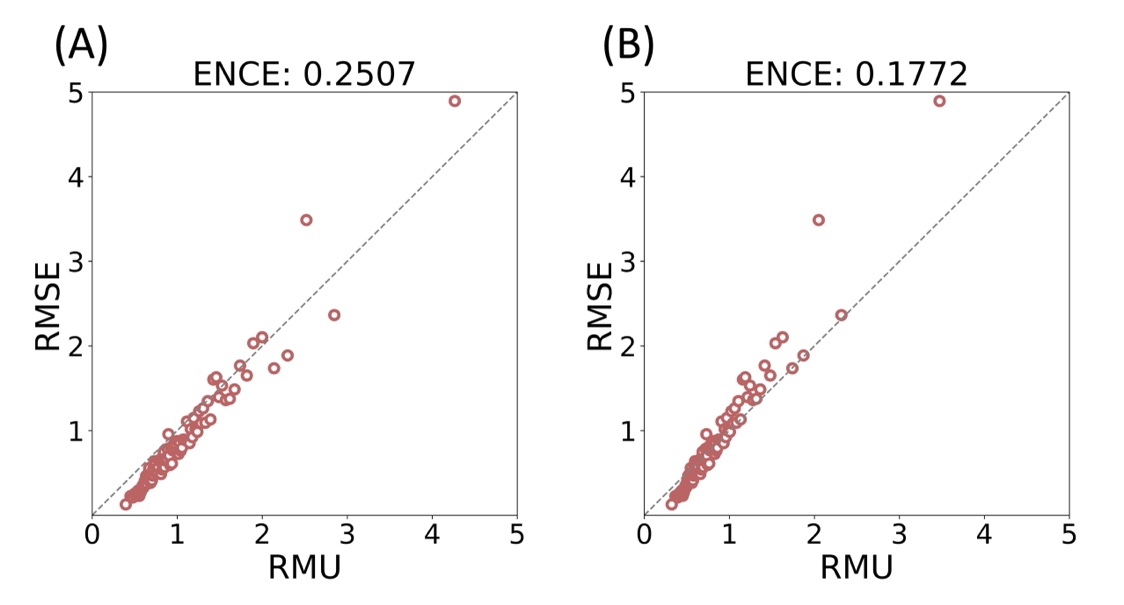


**Figure S11.** Error-based calibration curves and ENCEs for the QM9 testing set based on the aleatoric uncertainty calculated with (A) an ensemble of molecule-based uncertainty models and (B) an ensemble of molecule-based uncertainty models after *post-hoc* calibration.


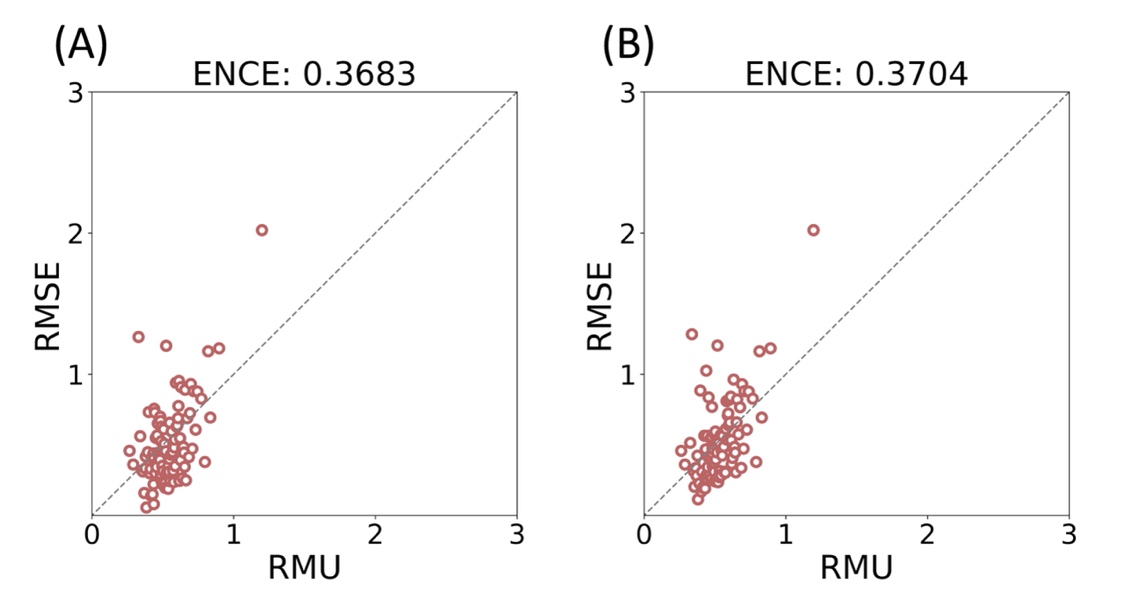


**Figure S12.** Error-based calibration curves and ENCEs for the Lipophilicity testing set based on the aleatoric uncertainty calculated with (A) an ensemble of atom-based uncertainty models and (B) an ensemble of atom-based uncertainty models after *post-hoc* calibration.


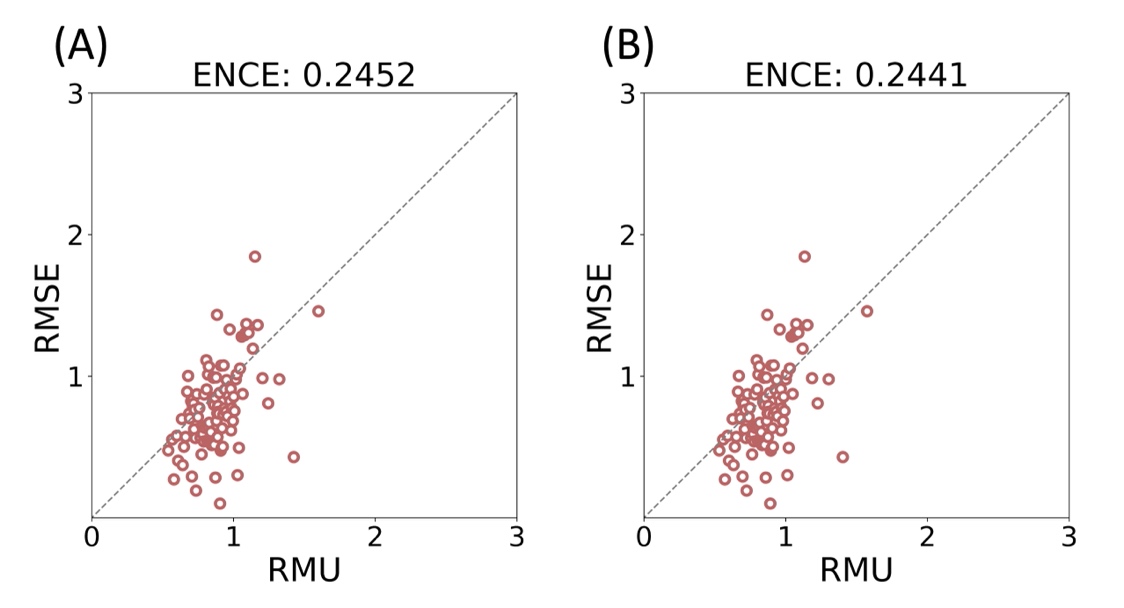


**Figure S13.** Error-based calibration curves and ENCEs for the Lipophilicity testing set based on the aleatoric uncertainty calculated with (A) an ensemble of molecule-based uncertainty models and (B) an ensemble of molecule-based uncertainty models after *post-hoc* calibration.


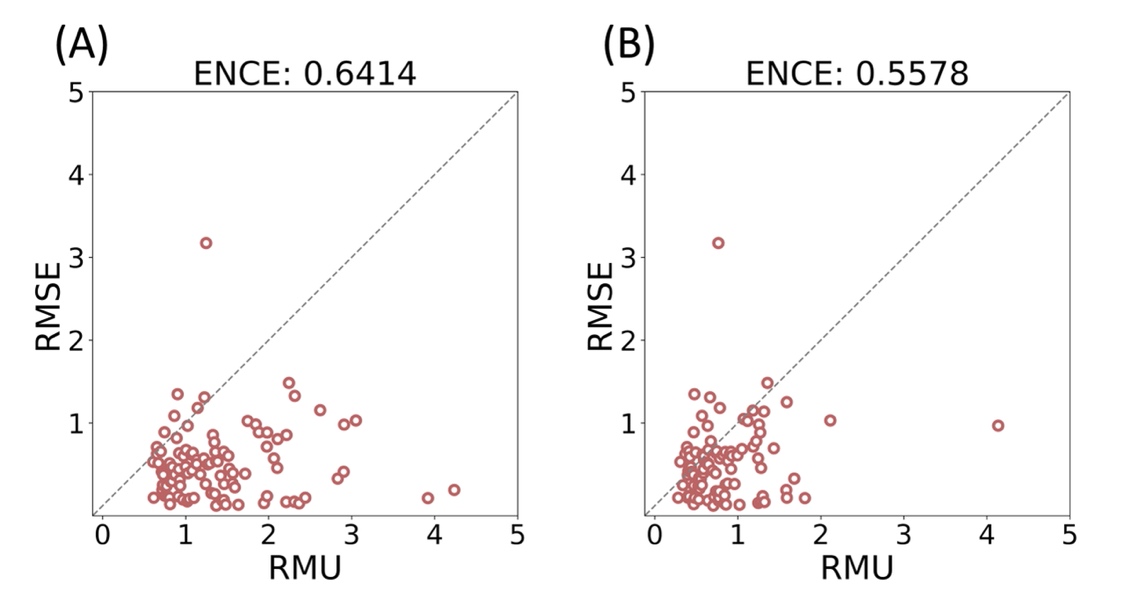


**Figure S14.** Error-based calibration curves and ENCEs for the Delaney testing set based on the aleatoric uncertainty calculated with (A) an ensemble of atom-based uncertainty models and (B) an ensemble of atom-based uncertainty models after *post-hoc* calibration.


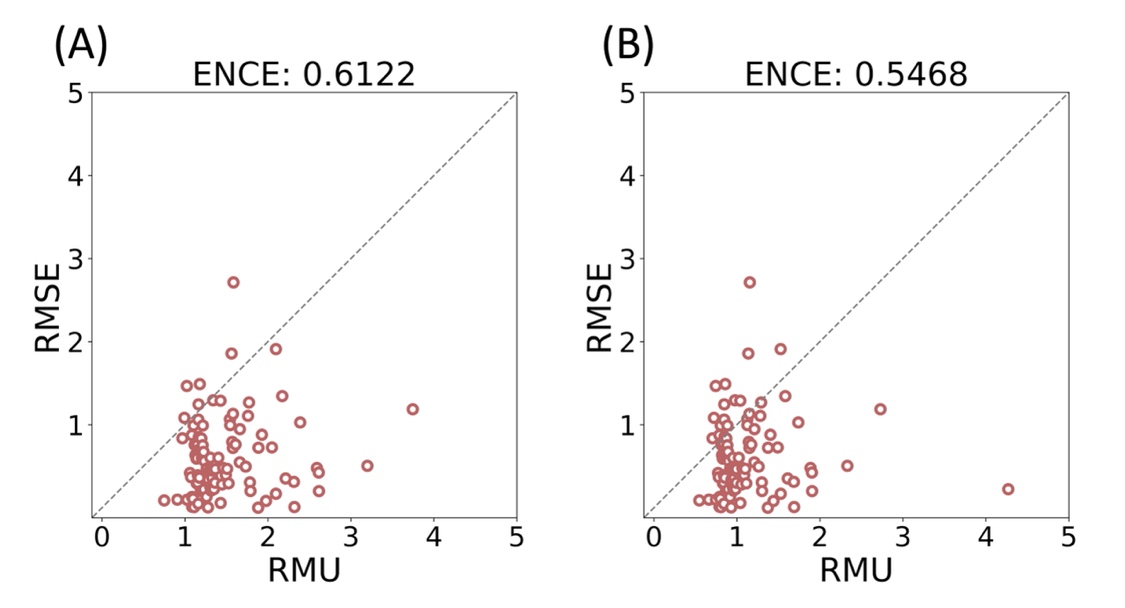


**Figure S15.** Error-based calibration curves and ENCEs for the Delaney testing set based on the aleatoric uncertainty calculated with (A) an ensemble of molecule-based uncertainty models and (B) an ensemble of molecule-based uncertainty models after *post-hoc* calibration.

**Correlation Coefficient Matrix**

The correlation coefficient matrix allows one to analyze how the model regards the correlations between atoms in a molecule. Here, the formation enthalpy of 2,2,2-trifluoroethylcyclopropane predicted by the atom-based uncertainty model is chosen as an example to illustrate this point. From the correlation coefficient matrix of the aleatoric uncertainty (Figure S16B), one can see that the model regards the carbon atoms in the cyclopropane groups (C:6, C:7, and C:8) to be highly correlated, which indicates that the three atoms reside in very similar chemical environments. A small molecular epistemic uncertainty (Figure S16C) suggests that the property values predicted by different models are consistent, implying that the ensemble model is confident about predicting the molecular property. However, the atomic epistemic uncertainties of the trifluoromethyl group are large, suggesting a discrepancy in the atomic contributions predicted by different models. Through the correlation coefficient matrix of the epistemic uncertainty, one can see that the atoms in the trifluoromethyl group (F:1, C:2, F:3, and F:4) show strong correlations with either positive or negative signs. The strong correlation suggests that a portion of the models in the ensemble attribute more property contributions to carbon atoms, while others attribute more to fluoride atoms. This situation mainly occurs when there are multiple ways to assign the atomic contribution values to predict the molecular property of interest, suggesting that a group-based property decomposition might be more suitable than the atom-based approach.


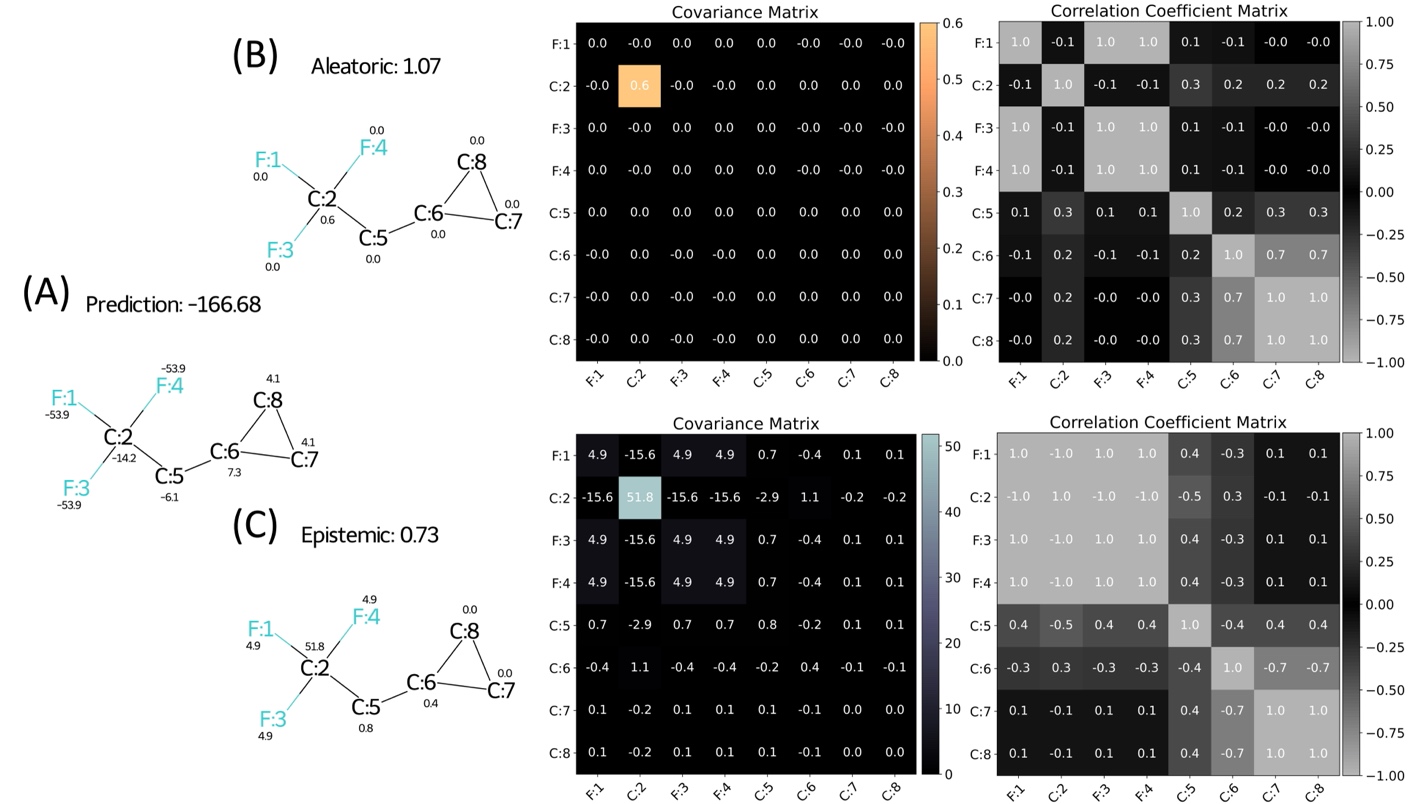


**Figure S16.** Formation enthalpy of 2,2,2-trifluoroethylcyclopropane predicted by the atom-based uncertainty model. (A) Predicted molecular enthalpy and the enthalpy value of each atom. (B) Aleatoric uncertainty with covariance matrix and correlation coefficient matrix. (C) Epistemic uncertainty with covariance matrix and correlation coefficient matrix.

**Initial Featurization**

Tables S1 and S2 list the differences between the atom and bond features used in Chemprop^1^ and our work. The new feature “in ring” represents whether the atom or bond is in a ring structure. The feature “in N-member ring” is a six-dimensional vector composed of binary bits to check whether the atom or bond is in the N-number ring, where $N\in\left\{ 3,4,5,\ldots8 \right\}$. For example, if a bond is in 4- and 7-member rings, the features will be [0, 1, 0, 0, 1, 0]. The formal charges, hybridization types, and bond types were removed in our model to avoid complications when multiple resonance structures are possible. The feature sets used for the atom- and molecule-based uncertainty models were the same to ensure a fair comparison between the models.

**Table S1.** Atom features used in Chemprop^1^ and this study.

| feature | description | size | Chemprop^1^ | this study |
| --- | --- | --- | --- | --- |
| atom type | atom type denoted by atomic number  (e.g. C is 6) | 100 | Yes | Yes |
| # bonds | number of bonds the atom connects  (H included) | 6 | Yes | Yes |
| chirality | unspecified, tetrahedral(CW/CCW rotation), or unrecognized chirality | 4 | Yes | Yes |
| # Hs | number of hydrogens the atom connects | 5 | Yes | Yes |
| atomic mass | mass of the atom, divided by 100 for scaling | 1 | Yes | Yes |
| in ring | whether the atom is a ring | 1 | No | Yes |
| in N-member ring | whether the atom is in an N-member ring (consider from 3- to 8-member ring) | 6 | No | Yes |
| formal charge | formal charge of the atom | 5 | Yes | No |
| hybridization | sp, sp2, sp3, sp3d, or sp3d2 | 5 | Yes | No |
| aromaticity | whether the atom is in an aromatic system | 1 | Yes | No |

**Table S2.** Bond features used in Chemprop^1^ and this study.

| feature | description | size | Chemprop^1^ | this study |
| --- | --- | --- | --- | --- |
| conjugated | whether the bond is conjugated | 1 | Yes | Yes |
| stereo | the stereochemistry of the bond  (none, any, Z, E, cis, or trans) | 6 | Yes | Yes |
| in ring | whether the atom is in a ring | 1 | Yes | Yes |
| in N-member ring | whether the atom is in an N-member ring (consider from 3-member to 8-member ring) | 6 | No | Yes |
| bond type | single, double, triple, or aromatic bond | 4 | Yes | No |

**Computational costs**

The computational cost mainly depends on **the size of the training set** and **the number of epochs the machine runs**. The wall times listed below are for training one atom-based uncertainty model. For instance, building an ensemble model containing five models would take five times the wall time listed below. The Delaney dataset contains 1128 molecules. We set 150 epochs to train but stop early if there is no improvement in 50 epochs. The QM9 dataset contains 134k molecules. We set 150 epochs to train but stop early if there is no improvement in 15 epochs. All timings were performed on eight cores of a 2.0GHz AMD EPYC Rome 64-core processor machine.

**Table S3.** Computational costs required to train one atom-based uncertainty model on the Delaney dataset.

| Epochs it ran | Time |
| --- | --- |
| 74 | 2 mins 47 secs |
| 81 | 3 mins 07 secs |
| 104 | 3 mins 57 secs |

**Table S4.** Computational costs required to train one atom-based uncertainty model on the QM9 dataset.

| Epochs it ran | Time |
| --- | --- |
| 61 | 211 mins |
| 85 | 286 mins |
| 108 | 366 mins |

**Analysis of Epistemic and Aleatoric Uncertainty**

Epistemic uncertainty of QM9 testing data calculated with the base model and nitrogen-noisy model are shown in Figure S17. Ideally, adding noise to the data of nitrogen-containing species should only affect the aleatoric uncertainty and leave the epistemic uncertainty unchanged. However, we found when using Deep Ensembles, the distribution of the estimate of epistemic uncertainty moderately increases in the nitrogen-noisy model (especially for the nitrogen-containing species). We believe that this is caused by the difficulty of fitting data with large noise. As data noise increases, finding the optimal fit for the samples becomes more challenging, and hence networks with different initial weights tend to reach different local minima, which leads to an increase in the discrepancy in the predictions, i.e., a higher estimate of epistemic uncertainty.

**
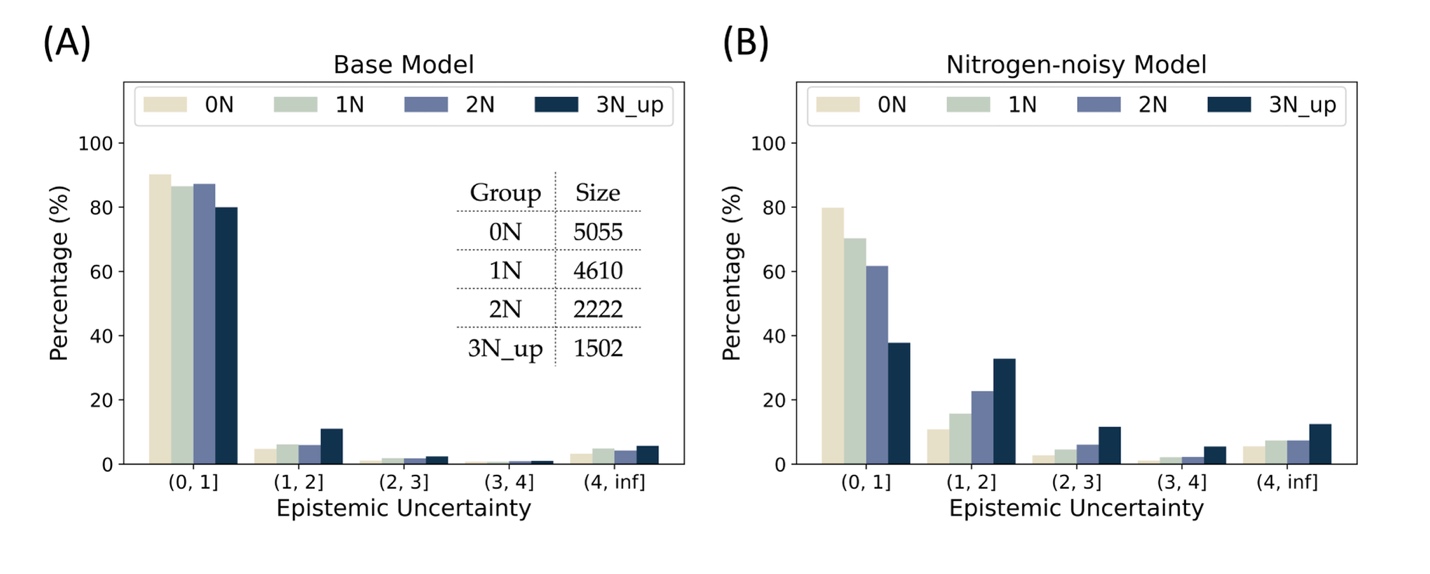
**

**Figure S17.** Epistemic uncertainty distributions of QM9 testing data calculated with (A) the base model and (B) the nitrogen-noisy model. The testing data are grouped by the number of nitrogen atoms in the molecule. The molecules without nitrogen atoms are denoted as 0N, molecules containing one nitrogen atom are denoted as 1N, and so on.

Aleatoric uncertainty of QM9 testing data calculated with the base model and nitrogen-ignorant model are shown in Figure S18. In principle, removing the data of nitrogen-containing species should only affect the epistemic uncertainty of nitrogen-containing molecules and should leave the aleatoric uncertainty unchanged. However, one can see a significant difference in the aleatoric uncertainty distribution between the base model and the nitrogen-ignorant model. Because the weights associated with the nitrogen atom are not trained in the nitrogen-ignorant model, the network outputs for nitrogen-containing species (including the estimate of aleatoric uncertainty) can be an arbitrary value and hence may result in a very different distribution of aleatoric uncertainty compared with the base model.

**
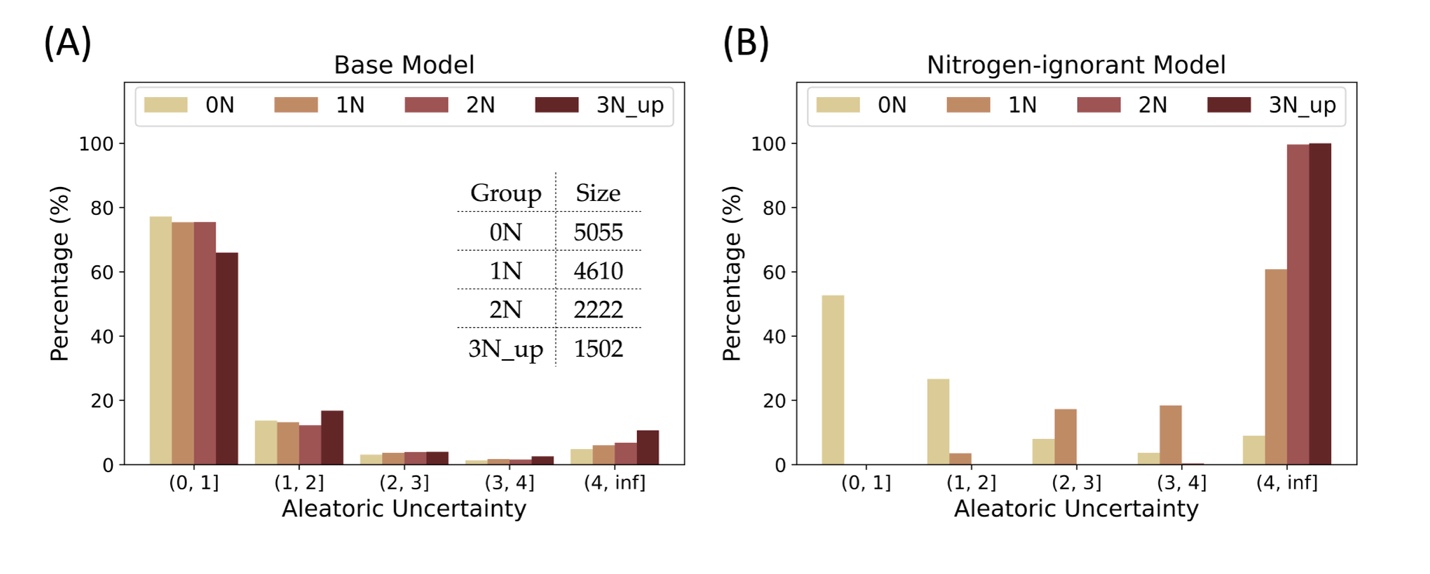
**

**Figure S18.** Aleatoric uncertainty distributions of QM9 testing data calculated with (A) the base model and (B) the nitrogen-ignorant model. The testing data are grouped by the number of nitrogen atoms in the molecule. The molecules without nitrogen atoms are denoted as 0N, molecules containing one nitrogen atom are denoted as 1N, and so on.

**Reference**

(1) Yang, K.; Swanson, K.; Jin, W.; Coley, C.; Eiden, P.; Gao, H.; Guzman-Perez, A.; Hopper, T.; Kelley, B.; Mathea, M.; Palmer, A.; Settels, V.; Jaakkola, T.; Jensen, K.; Barzilay, R. Analyzing Learned Molecular Representations for Property Prediction. J. Chem. Inf. Model. 2019, 59 (8), 3370–3388.
